# Behavioral nudges in social media ads show limited ability to encourage COVID-19 vaccination across countries

**DOI:** 10.1093/pnasnexus/pgae189

**Published:** 2024-08-06

**Authors:** Olgahan Çat, Jiseon Chang, Roman Hlatky, Huimin Li, Daniel L Nielson

**Affiliations:** Department of Government, University of Texas at Austin, Austin, TX 78712, USA; Department of Government, University of Texas at Austin, Austin, TX 78712, USA; Department of Political Science, University of North Texas, Denton, TX 76203, USA; Department of Government, University of Texas at Austin, Austin, TX 78712, USA; Department of Government, University of Texas at Austin, Austin, TX 78712, USA

**Keywords:** Covid, nudge, field experiment, social media, vaccination

## Abstract

Behavioral nudges in Facebook ads reached nearly 15 million people across six diverse countries and, consequently, many thousands took the step of navigating to governments’ vaccine signup sites. However, none of the treatment ads caused significantly more vaccine signup intent than placebo uniformly across all countries. Critically, reporting the descriptive norm that 87% of people worldwide had either been vaccinated or planned vaccination—social proof—did not meaningfully increase vaccine signup intent in any country and significantly backfired in Taiwan. This result contradicts prominent prior findings. A charge to “protect lives in your family” significantly outperformed placebo in Taiwan and Turkey but saw null effects elsewhere. A message noting that vaccination significantly reduces hospitalization risk decreased signup intent in Brazil and had no significant effects in any other country. Such heterogeneity was the hallmark of the study: some messages saw significant treatment effects in some countries but failed in others. No nudge outperformed the placebo in Russia, a location of high vaccine skepticism. In all, widely touted behavioral nudges often failed to promote vaccine signup intent and appear to be moderated by cultural context.

**Significance Statement**

Behavioral nudges offer simple interventions to move people toward positive personal and social actions at scale. How did they work across different countries during the Covid-19 pandemic? Online A/B testing assessed the effects of various advertising nudges compared to control in causing hits to governments’ vaccine signup sites. No nudge successfully increased link selection across all six countries. Highlighting vaccine efficacy negatively impacted signup clicks compared to placebo in Brazil and had no significant effects in all other countries. A charge to conform to an overwhelming majority failed in all six countries and backfired in Taiwan. No nudge worked in Russia. Such incongruities were common and suggest that nudges related to public health work very differently from place to place.

## Introduction

Minimizing death from communicable disease requires low-cost methods of promoting vaccination at scale. Rarely has this need been more acute than during the Covid-19 pandemic.

Widespread resistance to vaccines generates enclaves of unvaccinated people in which new disease variants can mutate, spread, and kill. And hesitancy appears to be common, with roughly one in five people surveyed in low- and middle-income countries expressing reluctance to be vaccinated (1). Given that Covid-19 vaccines have yet to penetrate majorities of populations in many lower-income countries, the need for scalable online solutions to reduce hesitancy remains urgent for Covid-19 and the next pandemic, whenever it comes.

Researchers are pursuing two broad approaches to the challenge. First, they seek to encourage those already inclined to vaccination. Interventions sending a variety of text reminders have effectively increased vaccine uptake for influenza and Covid-19 (2, 3). Early in the vaccination campaign, SMS reminders appear especially helpful if they induce a sense of ownership over the vaccine by noting that “a shot is waiting for you” (2) or urging the subject to “claim your dose” (3), but they appear to have no significant effects in later stages of the vaccine drive (4). Such studies have the added advantage of causal identification “in the wild”: as field experiments, they boast high external validity in terms of naturalism of settings, interventions, and outcomes.

Second, researchers aim to change the intentions of those who do not plan to be vaccinated. Plans may be malleable, a possibility reinforced by the facts that vaccine hesitancy has declined over time in almost every surveyed country and that vaccination rates continued to rise slowly many months after full vaccine availability. Some evidence has recently emerged that behavioral “nudges” in survey experiments describing broadly followed norms of conforming behavior, priming prosocial motives to protect others, and providing information on efficacy all prove effective in decreasing vaccine hesitancy measured as attitudes and self-reported intentions (5–10).

While the studies seeking to change vaccine intentions point to promising approaches, they are all survey experiments in which participants knew they were being studied. Possible Hawthorne effects, social desirability, or researcher demand may bias results (11–13). Moreover, researchers have long known that self-reported attitudes do not always correspond with observed behavior (14–16). Field experiments with greater ecological validity or naturalism are required to learn if effects uncovered through surveyed attitudes and self-reports translate to observable action.

To these ends, the research team designed a pre-registered experiment using A/B testing of ads on the Meta platform to encourage viewers to take the concrete step of navigating to their government’s vaccine signup website. In describing its A/B testing platform, Meta reports that it randomly assigns users to view different experimental versions of ads (17). However, researchers have worried that Meta platform A/B tests are subject to algorithmic bias due to their use of internal auctions (18). The research team sought to minimize algorithmic bias by setting the campaign to maximize reach, or the number of users’ feeds in which the ads appeared regardless of anticipated user actions. Postexperiment, the team sought to diagnose algorithmic bias, and little evidence suggests a threat to causal inference (see Section S7). However, in the absence of a complete description of the Meta randomization procedure, which the company withholds for proprietary reasons, questions remain about the internal validity of online A/B tests. This study might thus be viewed as a natural experiment in which the researchers do not control the randomization procedure but in which assignment of experimental conditions is effectively as-if random. In seeking to learn the effects of different ad messages on social media users’ online behavior, the present study has high naturalism or ecological validity at the possible expense of internal validity—a necessary tradeoff given the parameters of social media A/B testing.

The team crafted an array of advertisements with identical images but varying messages to learn if behavioral nudges drawn from social psychology and behavioral economics would promote greater vaccine uptake generally across different countries. All of the nudges were compared to a placebo ad that contained the image and the signup link but not the encouragement text. The ads were randomly assigned within groups of five alternative experimental conditions, as described below. Importantly, the experiments test substantively identical messages across multiple country contexts, which present variable background conditions and therefore may produce heterogeneous effects cross-nationally (19).

Interventions reporting descriptive norms of what most people actually do have robustly increased desired actions across a wide array of behaviors (5, 6, 9, 20–22). In the context of the Covid-19 pandemic, Van Bavel et al. predict that “[p]roviding accurate information about what most people are doing is likely to be helpful if what most people are doing is desirable (health-promoting)” (10, 463). While promising survey experimental evidence suggests that such a “social proof” intervention could increase vaccination in multiple countries (6, 8, 9), to our knowledge it has not been tested in an experiment with high ecological validity. Accordingly, the research team deployed an ad stating that “87% of people have been vaccinated or plan to get vaccinated (according to polls by Morning Consult).” The ad suggested an overwhelming norm and therefore should have provided strong social proof enjoining vaccination.

Additionally, reminding people that their actions can have positive effects on others—priming prosocial motivation—appears to increase vaccination intentions across studies in multiple countries (6, 7), but with questions about robustness (9). The present study primed subjects’ interest in helping others with two distinct messages: “Protect lives in your family” and “Protect lives in your community.” The research team pre-registered the expectation that the “protect family” message would have the strongest treatment effects cross-nationally. Both prosocial messages can be directly compared to an additional treatment condition deployed to prime self-interest: “Protect your life.”

Further, interventions providing information about vaccine efficacy have increased inoculation intentions reported in surveys across different countries (7), though not necessarily robustly (9). The present study attempted to signal the effectiveness of the vaccines in multiple ways. The first efficacy intervention stated, “Vaccination is 96% effective against hospitalization (including from the Delta variant, according to a study by Public Health England).” A second efficacy intervention associated the vaccines with science: “Follow medical scientists.” Additional efficacy signals were sent by identifying the vaccines as produced in different countries known for strong scientific expertise: Germany and the United States. However, it is worth emphasizing that these country treatments also associate the vaccines with other factors—some probably negative—that might be connected to these countries in the minds of subjects.

## Results

**Fig. 1.**
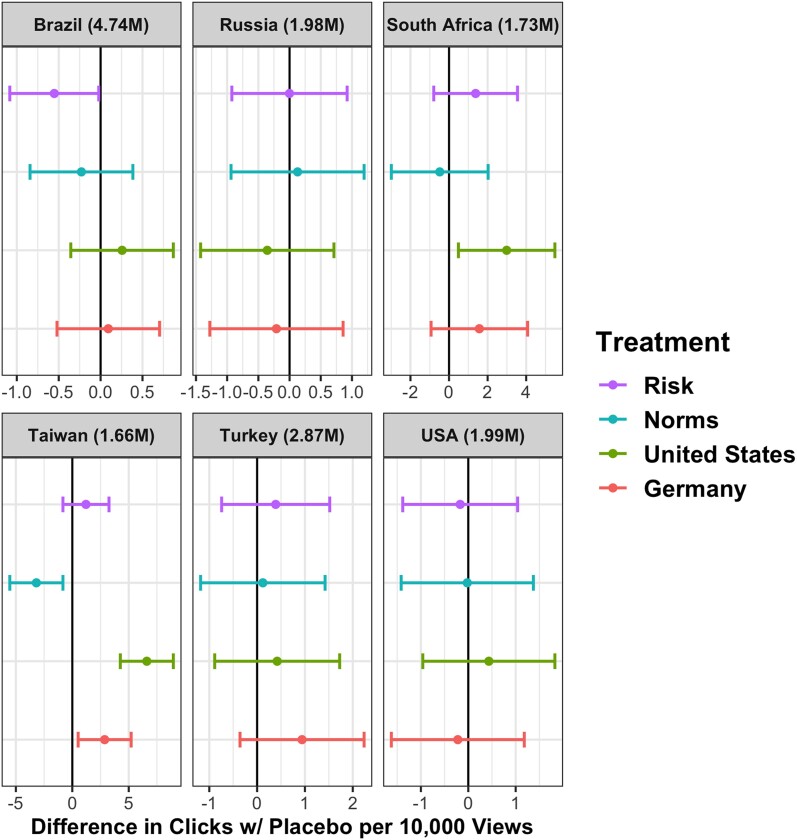
Group A treatments.

**Fig. 2.**
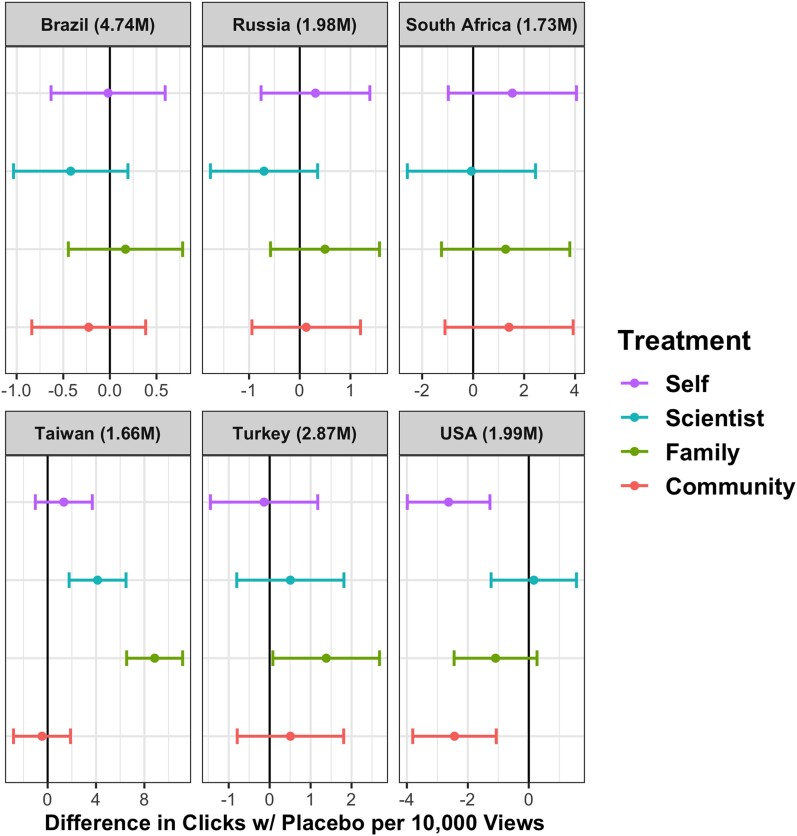
Group B treatments.

Between October 2021 and January 2022, the ads appeared in the feeds of 14,930,000 Meta Users in six countries: Brazil, Russia, South Africa, Taiwan, Turkey, and the United States. The vaccination rates in the countries varied cross-nationally and over time during the experiments as seen in Table S15. The number of clicks per view varied between 18 and 192 per 100,000 user views or reach, which is Meta’s term for the ads’ appearance in users’ feeds. To measure the effect of each treatment, we employed ordinary least squares (OLS) models in which the unit of analysis is each ad shown. The dependent variable is whether the signup link was clicked, and the main independent variable is treatment compared to placebo as the baseline condition. Given that we published ads in two or three rounds per country, we include dummy variables for rounds in all models. Figures 1 and 2 show the effect of each treatment across all countries.^a,b^ Unadjusted confidence intervals indicated by the horizontal lines are set at 0.95.

The nudge priming descriptive norms through the provision of social proof (“Norms” in Fig. 1)—i.e. the ad stating that 87% of people are already or plan to be vaccinated—caused a significant decrease in vaccination signup clicks in Taiwan compared to placebo (P=0.007). In all other countries, the social proof treatment was statistically indistinguishable from placebo, with relatively precisely estimated nulls.

The prosocial motivation treatments (“Family” and “Community” in Fig. 2) produce varying results depending on the country context. The admonition to “protect lives in your family” significantly increased signup clicks compared to placebo in Turkey (P=0.037) and Taiwan (P=0.000). In South Africa, the message to protect family did not outperform the charge to “protect lives in your community” and was statistically indistinguishable from placebo. In the United States, the protect-family ad results were null compared to placebo. The protect-community ad (P=0.000) and encouragement to “protect your life” (P=0.000) (“Self” in Fig. 2) significantly decreased signup clicks in the United States compared to the placebo. In Brazil, the self-interest encouragement, family, and community ads were all null compared to placebo. The “Self” nudges did not produce statistical differences from placebo in either Turkey or Russia.

The efficacy treatment signaling reduced hospitalization risk (“Risk” in Fig. 1) performed worse than placebo in Brazil (P=0.04). In all other countries, the effects of this reduced-risk message were statistically indistinguishable from placebo. This provides additional evidence of treatment heterogeneity across country contexts, and it suggests that psychological responses to behavioral stimuli may be culturally contingent or moderated by other local conditions (23).

The efficacy treatment associating vaccines with science, “Follow Medical Scientists,” significantly increased signup clicks in Taiwan (P=0.000) compared with the placebo. The scientists nudge decreased click rates in Brazil (P=0.17) and Russia (P=0.19), although not at a statistically significant level. “Scientists” produced precisely estimated nulls elsewhere. The remaining efficacy treatments noted two of the principal countries where the vaccines were produced and which have established reputations for scientific expertise, the United States and Germany. Figure 2 indicates that Germany caused more clicks for sign up in Taiwan (P=0.016). The US treatment increased clicks for sign ups in South Africa (P=0.019) and Taiwan (P=0.000). The country treatments produced precisely estimated null results in other contexts at conventional significance levels.

## Discussion

In this article, we investigated whether various encouragement messages, focusing on interventions related to social proof, prosocial nudges, and vaccine efficacy/credibility, bolstered individual vaccination signup clicks. In the study, few individuals chose to click the signup links, and most of the persuasive messages had limited effects. Yet, in some countries, certain messages did significantly motivate users—and in a few countries, some messages backfired and had significant demotivating effects.

The most surprising finding involved the evocation of social proof, or information reporting descriptive norms about how overwhelming majorities of other people behave. As noted, social proof interventions have produced strong effects in the lab and field across many prominent studies (5, 6, 20–22, 24, 25). However, on the topic of Covid vaccination, some evidence already suggested the probable ineffectiveness of the social-proof nudge (7). However, a recent synthesis of evidence indicated overall small but significant effects in real-world settings, though most reviewed studies were lab, survey, or other controlled experiments; the one field study produced a null finding (9). The results here reinforce the null findings in the field. In no case did social proof positively and significantly motivate navigation to vaccine signup sites. The findings here suggest that descriptive social norms do not encourage conforming behavior in any of the tested country contexts and, in the case of Covid-19 vaccination, may even backfire.

Indeed, in Taiwan, reporting that 87% of people had been or planned to be vaccinated backfired significantly. The negative treatment effect is relatively large in substantive terms and highly significant statistically (P=0.007), a level that survives multiple-comparisons adjustments (see Table S1). Our study was not designed to identify the relevant cross-country factors that explain why some messages work and why others backfire. The selected countries differ not only in culture and values but also in geography, politics, and economics—not to mention vaccination rates. We chose our country sample precisely for these variations—with the hope that some messages would survive this rigorous test of external validity. However, given these variations, we cannot definitively identify why the social proof treatment backfired in Taiwan. Nonetheless, we speculate that high pre-existing vaccination rates may have played a role. During the fielding of the experiment, close to 75% of Taiwanese had received at least one dose of the vaccine due to a sustained government information campaign that started in May 2021. The campaign focused heavily on societal responsibility, and experts suggest a norm of “cooperating with the government in national emergencies as a civic responsibility and shar[ing] the recognition that everyone is in this together” is strongly held in Taiwan (26). As such, it is possible that the social proof treatment backfired due to its lack of novelty and its reinforcement of an already internalized norm.

The failure of social proof to move Meta users toward signup for vaccination in any country—and to even backfire—questions the generalizability of nudges relying on descriptive social norms for causing conformity in the critical domain of health behaviors. More research will be needed to demarcate the limits of nudges using social proof and to identify what factors may produce the backfire effect we saw in Taiwan. The social-proof result is especially interesting in light of a recent synthesis of evidence regarding Covid-19 interventions (9). The findings here suggest reason for skepticism that descriptive norms function to motivate vaccination generally.

We also investigated the persuasive potential of prosocial messages. We focused on the benefits vaccination could bring to an individual’s family and community, respectively. We compared the performance of these messages to a message that focuses on the benefits vaccination has for individual well-being. In other words, we sought to determine whether messages priming self-interest can also motivate vaccination, and whether these messages are more effective at doing so than prosocial messages. We find substantial heterogeneity across the various countries.

Prosocial messages focusing on the family seem to be an effective encouragement in two countries: Taiwan and Turkey. While the results in these two countries contrast with a recent evidence synthesis, the nulls in the other four countries generally reinforce the prior conclusions (9). In Taiwan, for example, the family treatment promoted more engagement than any other condition. Here, we speculate that the message activated strong norms related to filial responsibility and collective well-being, which previous research has shown to have downstream consequences on various prosocial behaviors including vaccination (27, 28). Similarly, in Turkey, the family treatment attracted more clicks than the placebo condition. Prosocial messages have been hypothesized to positively predict vaccine uptake or provaccination attitudes in recent studies in Turkey, lending further, confirmatory evidence to our results (29–32).^c^ Messages focused on protecting one’s own community had limited effects. Moreover, these calls actually backfired in the United States. Here, we suggest that the distal, abstract nature of the referent—“community”—led to limited engagement.

Our United States findings also challenge previous work. Existing findings suggest that prosocial messages focusing on proximate social groups, like the family, increase vaccination intentions amongst Americans (33, 34), and that these effects are often stronger than those associated with “selfish” motivations like self-protection (35–37). However, again a recent evidence synthesis casts doubt on these conclusions (9). This study’s findings suggest that prosocial messages, especially those that focus on family, may be effective at bolstering vaccine uptake in multiple, culturally different locales. Moreover, focusing on individual self-interest seems to be generally ineffective, which aligns with the recent evidence synthesis (9). Our results suggest that calls to protect one’s family, one’s community, or oneself have limited influence—if not backfire potential—on engagement. This suggests that, in the wider global context, prosocial messages may actually do more harm than good, perhaps unless strong norms related to collectivism or family are present in a given country context. Finally, we note that messages with calls to protect oneself were generally ineffective at spurring engagement and even backfired in the United States.

We investigated the impact of vaccination efficacy through three different message types. First, we employed messages that directly suggested vaccination reduces hospitalization risk by 96%. This message failed to produce positive effects in any of the countries and, in Brazil, it even backfired (though at marginal levels of statistical significance). These results underscore that even messages that included a scientific citation are easily discounted by audiences and may even activate skepticism in especially mistrustful sub-populations.

Second, we considered whether associating vaccines with medical scientists could bolster persuasive impact. Here, we assumed that adding expert credibility would prove effective; yet, this message did little to shift individual engagement anywhere except in Taiwan, where it increased signup clicks significantly. Finally, we manipulated messages to include information about a vaccine’s country of origin. Here, we found that South African and Taiwanese respondents were more responsive to information that vaccines are produced in the United States. Moreover, Taiwanese respondents also responded positively to messages about German vaccine production. We suggest that these messages offered signals of vaccine quality in countries that—at least in the early stages of the pandemic—had to either rely on alternative sources or had trouble procuring vaccines in general. To provide further evidence, future research could focus on whether Chinese- or Russian-made vaccines evoke different reactions. Nonetheless, these results indicate that indirect signals of vaccine quality may be effective at bolstering engagement in contexts where high-quality vaccines may have been difficult to procure.

Taken together, these results suggest that widely touted informational nudges have limited effects when it comes to motivating vaccination. Moreover, in some cases, these nudges appear to backfire. Drawing on previous work, we have attempted to speculate on the possible reasons for the observed effects. However, various factors—ranging from culture, to politics, to economics, to geography—can potentially play a role in explaining the statistical significance and direction of the various treatments. Our hope is that future research builds on these findings and investigates the reasons underlying this treatment heterogeneity.

This treatment heterogeneity across country contexts is itself a striking result of the field experiment. Often, researchers tout the external validity of field experiments, especially as it relates to their naturalism. Our design, which used Meta to achieve a high degree of realism, led to results that underscore an additional, critical element of external validity: context. The effects of provaccination nudges varied notably across the six countries under study. This variation suggests that the respective country contexts may moderate—if not mediate—the effects of these nudges on vaccine uptake. Thus, when crafting provaccine persuasive messages, the best strategy may be to incorporate norms, data, and calls-to-action that take country-specific factors into account. Ultimately, decreasing vaccine hesitancy remains an essential challenge in the post-Covid era, and our results provide a first glance at which strategies may or may not be effective across diverse countries.

## Methods

### Research design and data

This research received approval from and was determined exempt by the University of Texas at Austin’s Institutional Review Board (STUDY00001109). No individual-level data were seen by researchers, only aggregate statistics. Any individually identified data were known only to the Meta social media platform. Because all interactions were mediated by the platform and no individual-level data were conveyed to researchers, informed consent was not sought and the IRB approved the field experiment on that basis.

The experiment uses A/B testing with multiple conditions. Based on a binary outcome, A/B testing assesses the difference between the proportions of two options: A and B (38). A/B testing compares two or more ads to determine which version performs best. Our experiment employs Meta’s A/B testing platform to probe the effects of messages embedded with behavioral nudges. The subjects’ intention to receive Covid-19 vaccination is measured using their clickthroughs to vaccine signup sites. Meta reports that its A/B testing platform divides the budget equally, randomly exposes users to each version of the ads, and provides statistically comparable results. For this experiment, we use the A/B testing feature by changing the primary text in the ad creative to encourage Covid-19 vaccination.^d^

The ads include the following: page name, treatment statement, image, click statement, and a website URL. In all six countries, the page name was kept consistent “Information Sharing Project,”^e^ to ensure that the page name did not affect the outcomes. A total of eight treatments were randomly assigned to individuals along with one placebo in two groups of five conditions each.

Group A included the placebo, the Risk treatment—“Vaccination is 96% effective against hospitalization (including from the Delta variant, according to a study by Public Health England),” the Norms (social proof) treatment—“87% of people have been vaccinated or plan to get vaccinated (according to polls by Morning Consult),” and the United States and Germany treatments—“One widely used vaccine has been developed in the [United States/Germany].” Group B included the placebo, the Self-interest treatment—“Protect Your Life,” the Scientist treatment—“Follow Medical Scientists,” the Family treatment—“Protect Lives in Your Family,” and the Community treatment—“Protect Lives in Your Community.” For each group of four treatments and placebo, Meta randomly assigned one of the five ads from the set to each user feed in the study.

The meaning of the experimental conditions was kept consistent across all countries and was translated into each country’s local language, if English was not a dominant language.^f^ Each combination only varies one condition—the treatment messages—enabling the results to indicate which treatments are effective with all other confounding factors held constant in expectation.

In the ads,^g^ we show a statement “Click here to sign up for the vaccine” with a button stating “Learn More” and an image that is constant within each country. The research team selected images that depicted a happy, vibrant, and “normal” life, which had been difficult during Covid-19. We measure the number of interactions with a given ad, in particular the number of clicks on the link leading to an actual government-sponsored vaccination signup page. For the website URL, we included the government web address where users could sign up to receive vaccinations in each country.^h^

We conducted the experiment across six countries: Brazil (São Paulo),^i^ Russia, South Africa, Taiwan, Turkey, and the United States. The diverse set of countries should help to alleviate external validity concerns and enable assessment of cross-country heterogeneity in treatment effects. Using the “custom audience” feature in Meta ads, each experiment targeted the country in question. For instance, a message in Turkish would be set to target the population of Meta users in Turkey. Recruiting samples from these six countries ensures meaningful geographical, cultural, and socio-economic variation. In addition, the countries were selected for three main reasons. First, citizens in the selected countries can freely use Facebook/Instagram. Second, in a study in which biases and prejudices play a role, nuances matter, and these are countries where the research team had a strong command of the native languages. Thus, all of the ads used in the study were vetted by native speakers. Third, at least one of the vaccines in our study was widely available in each country.

When implementing A/B testing using Meta ads, we built upon Orazi and Johnston (39). Predating the Facebook A/B testing feature, Matz et al. (40) conducted experiments on Facebook comparing different types of advertisements, which were criticized for the lack of random assignment (41). Previously, Facebook experiments had drawn fire because the platform did not have tools to ensure random assignment prior to November 2017 and thus had the potential to introduce endogenous variation (39, 41–43). The post-2017 procedure addresses that inference challenge (39), though concerns about algorithmic bias persist (18). It seems likely that A/B tests set to maximize interactions, link clicks, or sales will engage the internal auction in ways that may induce algorithmic bias because the algorithm is predicting user actions in these cases.

However, A/B tests set to maximize “reach” as the objective—in which the ads appear in the maximum number of user feeds without regard to anticipated user behavior, as in this study—have not been directly considered by critics (18). Maximizing reach should minimize algorithmic bias because the assignment procedure ought to be orthogonal to predicted user actions (39). In postexperiment diagnostics, we employed a general independence test for two sets of variables. We find little evidence that the Meta algorithm maximizing reach induced threats to causal inference (see Section S7).

During the initial rounds of experiments, in the interest of statistical power to detect small differences in treatment effects, we targeted 100 link selections for each condition for each country.^j^ However, because we encountered greater heterogeneity across countries in click rates per ad spend than anticipated, we adjusted the amounts spent for each ad buy for each country and added advertising rounds for each country as needed to approximate the target number of link clicks. We conducted the experiment in two or three rounds for each country: two rounds in Turkey, Russia, and Taiwan, and three rounds in the United States, Brazil, and South Africa.^k^ As seen in Tables S9–S14, there was general—though far from complete—consistency for experimental conditions across rounds in each country, most of which might reasonably be accounted for by random variation. Because Meta, for proprietary reasons, does not reveal the technical details applied in its A/B testing platform, it is difficult to discern the source of the remaining anomalies within conditions across rounds.^l^ We have elected to report the results as received and anticipate that future studies might answer key remaining questions.

### Outcomes and statistical analysis method

Meta does not provide any individual-level data, thus we use the behavioral outcomes, clicks on the provided signup link, to measure the effectiveness of treatments. We compare differences in clicks by employing an OLS regression model. Our primary outcome measure of interest is clicks—whether each individual selected or not the “Learn More” link in the advertisement. Our main independent variable is treatment compared to placebo, and our control variables are binary indicator variables marking each round of the experiment. We checked robustness of these tests using the nonparametric technique of randomization inference (44), which is permutation-based inference and equivalent to Fisher’s permutation, and results can be seen in Section S2 and Figs. S3–S50. Permutation-based inference did not produce substantively different findings to those presented from OLS.


(1) $${\text{Clicks}}_{i}=\beta_{0}+\beta_{1}{\text{Treatment}}_{i}+\beta_{2}{\text{Round}}_{i}+u_{i}$$

## Supplementary Material

pgae189_Supplementary_Data

## Data Availability

Replication data will be deposited to the Open Science Framework, where the project was pre-registered upon publication. The code will be deposited to the Open Science Framework.

## Notes

a Coefficient estimates for each treatment and country are in Tables S1 and S2. Section S6 shows ad placements by country and by platform. Tables S3 and S4 show results from Instagram only. Tables S5 and S6 show if there was a difference compared to the placebo regarding positive or negative reactions.

b Results for permutation-based randomization inference (RI) are presented in Section S2. We also account for multiple comparisons with Holm and Benjamini–Hochberg (BH) adjustments. Holm adjustment, with its sequential approach, controls for the family-wise error rate (FWER) more strongly relative to the Bonferroni and BH adjustments which focus on accounting for the false discovery rate (FDR) (45).

c Though, one study does find that “protect yourself” messages are more effective than “protect your family” or “protect your country” messages in Turkey (46).

d
https://www.facebook.com/business/help/1738164643098669?id=445653312788501

e The page name in the United States was “University of Texas Information Sharing Project,” while for the other countries, we named the pages either “Information Sharing Project” or a name equivalent to that in the countries’ languages.

f For Brazil, the treatments were translated into Portuguese; for Russia, into Russian; for Taiwan, into Traditional Chinese; and for Turkey, into Turkish. In South Africa, we used English for the treatment messages, since it is one of the country’s many official languages and is widely learned as a second language. This choice was to ensure that the treatment messages were understood by a large number of people, as English serves as a common linguistic bridge among South Africa’s diverse population, and so that we did not signal ethnic differences through the message.

g Facebook ads for all countries are shown in Section S5.

h URL links can be found in Section S8.

i In the case of Brazil, we targeted São Paulo, the largest city in Brazil, because the vaccination signup websites were different for each Brazilian region. This geographic specificity helped to avoid confusion among users.

j We took a consistent approach and achieved or exceeded the target click rate in all countries except Russia. Due to the disproportionately high monetary cost of link clicks coupled with insufficient evidence of treatment effects, we made the decision to conclude the experiment in Russia after two rounds.

k Details of the link clicks and reach by treatment and country are in Section S3 and data for each round are provided in Tables S9–S14.

l The most glaring is the “Follow Medical Scientists” result in the United States, which showed an order of magnitude difference in Round 2 from placebo but was comparable to placebo in Rounds 1 and 3.

## Preprints

A preprint of a previous version of the article is published at https://doi.org/10.21203/rs.3.rs-2319578/v1.
