# Supplementary material for: Behavioral nudges in social media ads show limited ability to encourage COVID-19 vaccination across countries
Source: PNAS Nexus. 2024 Aug 6;3(8):pgae189. doi: 10.1093/pnasnexus/pgae189 (PMC11302845; doi:10.1093/pnasnexus/pgae189)
Supplement: pgae189_Supplementary_Data [file pgae189_supplementary_data.pdf]

Supplemental Information

1. Difference in Clicks

Table S1. Group A: Difference in Clicks w/ Placebo per 10,000 Views

Country	Treatment	Estimate	Unadjusted p-value	Holm	BH
Brazil	United States	0.25644842	0.41138655	1	0.62078591
Brazil	Germany	0.09210339	0.76809753	1	0.76809753
Brazil	Risk	-0.5527095	0.04117842	0.16471366	0.16471366
Brazil	Norms	-0.2284474	0.46558943	1	0.62078591
Russia	United States	-0.3574665	0.51193983	1	0.99800287
Russia	Germany	-0.210378	0.69939163	1	0.99800287
Russia	Risk	-0.0011799	0.99800287	1	0.99800287
Russia	Norms	0.12879081	0.81285954	1	0.99800287
South Africa	United States	2.98716062	0.01924106	0.07696425	0.07696425
South Africa	Germany	1.57034267	0.21858717	0.63949793	0.29144956
South Africa	Risk	1.37882343	0.21316598	0.63949793	0.29144956
South Africa	Norms	-0.4818911	0.70648821	0.70648821	0.70648821
Taiwan	United States	6.60427824	0.00000004	0.00000015	0.00000015
Taiwan	Germany	2.86662157	0.01670032	0.03340064	0.02226709
Taiwan	Risk	1.2104612	0.24544639	0.24544639	0.24544639
Taiwan	Norms	-3.1841927	0.00798187	0.02394561	0.01596374
Turkey	United States	0.41934022	0.52923823	1	0.70565098
Turkey	Germany	0.94001373	0.15562372	0.62249486	0.62249486
Turkey	Risk	0.38837691	0.50048048	1	0.70565098
Turkey	Norms	0.12004756	0.8565394	1	0.8565394
United States	United States	0.43400524	0.54152059	1	0.97625661
United States	Germany	-0.2202301	0.75819557	1	0.97625661
United States	Risk	-0.1701036	0.78321268	1	0.97625661
United States	Norms	-0.0211832	0.97625661	1	0.97625661

Draft

Table S2. Group B: Difference in Clicks w/ Placebo per 10,000 Views

Country	Treatment	Estimate	Unadjusted p-value	Holm	BH
Brazil	Family	0.16749853	0.59216721	1	0.78955628
Brazil	Community	-0.2269481	0.46637885	1	0.78955628
Brazil	Scientist	-0.419962	0.17981754	0.71927015	0.71927015
Brazil	Self	-0.0188398	0.95187775	1	0.95187775
Russia	Family	0.49895233	0.36329646	1	0.72659292
Russia	Community	0.12749719	0.81545	1	0.81545
Russia	Scientist	-0.7052231	0.19130796	0.76523183	0.72659292
Russia	Self	0.30966654	0.57156922	1	0.76209229
South Africa	Family	1.27497491	0.32072685	0.92151814	0.4276358
South Africa	Community	1.40915644	0.27205443	0.92151814	0.4276358
South Africa	Scientist	-0.067349	0.95812612	0.95812612	0.95812612
South Africa	Self	1.53928374	0.23037954	0.92151814	0.4276358
Taiwan	Family	8.85055258	0.00000000	0.00000000	0.00000000
Taiwan	Community	-0.4654264	0.69875421	0.69875421	0.69875421
Taiwan	Scientist	4.13126398	0.00057822	0.00173465	0.00115643
Taiwan	Self	1.33706856	0.26564034	0.53128067	0.35418712
Turkey	Family	1.38142564	0.03776153	0.15104613	0.15104613
Turkey	Community	0.50816117	0.44357797	1	0.59857134
Turkey	Scientist	0.50561715	0.44892851	1	0.59857134
Turkey	Self	-0.1362996	0.83854405	1	0.83854405
United States	Family	-1.0901024	0.11607829	0.23215659	0.15477106
United States	Community	-2.4371257	0.00049035	0.00147104	0.00098069
United States	Scientist	0.1647369	0.8176865	0.8176865	0.8176865
United States	Self	-2.6301332	0.00014185	0.00056741	0.00056741

Table S3. Group A: Difference in Clicks w/ Placebo per 10,000 Views (Instagram)

Country	Treatment	Estimate	Unadjusted p-value	Holm	BH
Brazil	United States	0.11187325	0.70451884	1	0.9522518
Brazil	Germany	0.02727162	0.92600156	1	0.9522518
Brazil	Risk	-0.3184085	0.21203082	0.84812327	0.84812327
Brazil	Norms	-0.0176337	0.9522518	1	0.9522518
Russia	United States	-0.5129101	0.25990221	0.77970663	0.50233898
Russia	Germany	-0.2394199	0.59927255	0.77970663	0.59927255
Russia	Risk	-0.6351506	0.10738733	0.42954932	0.42954932
Russia	Norms	-0.4025555	0.37675423	0.77970663	0.50233898
South Africa	United States	0.28621308	0.83254871	1	0.92550519
South Africa	Germany	1.37975333	0.2931628	1	0.5863256
South Africa	Risk	0.10871967	0.92550519	1	0.92550519
South Africa	Norms	1.56832898	0.26683381	1	0.5863256
Taiwan	United States	-1.2159796	0.26936332	0.53872664	0.35915109
Taiwan	Germany	0.52287498	0.57992051	0.57992051	0.57992051
Taiwan	Risk	-1.2865116	0.13493457	0.40480371	0.26986914
Taiwan	Norms	-1.8310985	0.04970671	0.19882685	0.19882685
Turkey	United States	0.42579376	0.51884186	1	0.71279417
Turkey	Germany	0.94466461	0.14961033	0.59844131	0.59844131
Turkey	Risk	0.3540686	0.53459562	1	0.71279417
Turkey	Norms	0.1445654	0.82609976	1	0.82609976
United States	United States	-2.4421439	0.05596734	0.22386935	0.22386935
United States	Germany	-0.6705702	0.58675244	1	0.78233658
United States	Risk	-0.6810734	0.52006021	1	0.78233658
United States	Norms	0.17401949	0.88742273	1	0.88742273

Draft

Table S4. Group B: Difference in Clicks w/ Placebo per 10,000 Views (Instagram)

Country	Treatment	Estimate	Unadjusted p-value	Holm	BH
Brazil	Family	-0.0957434	0.74479628	1	0.85040451
Brazil	Community	-0.1987534	0.49720577	1	0.85040451
Brazil	Scientist	-0.3527254	0.23331288	0.93325151	0.85040451
Brazil	Self	-0.0554365	0.85040451	1	0.85040451
Russia	Family	-0.2017303	0.66390219	1	0.88520291
Russia	Community	-0.5045965	0.27225899	1	0.88520291
Russia	Scientist	-0.3452163	0.44266585	1	0.88520291
Russia	Self	0.05947725	0.89706342	1	0.89706342
South Africa	Family	4.57016512	0.00294694	0.01178774	0.01178774
South Africa	Community	1.7212204	0.23022833	0.46045666	0.30697111
South Africa	Scientist	3.09457546	0.02721756	0.08165268	0.05443512
South Africa	Self	-0.2777612	0.83235817	0.83235817	0.83235817
Taiwan	Family	1.08654825	0.32516559	0.97549678	0.4884455
Taiwan	Community	-0.7854105	0.4232556	0.97549678	0.4884455
Taiwan	Scientist	-1.3692269	0.15987546	0.63950183	0.4884455
Taiwan	Self	0.70538265	0.4884455	0.97549678	0.4884455
Turkey	Family	1.35314744	0.039708	0.15883198	0.15883198
Turkey	Community	0.53029032	0.41973452	1	0.60770091
Turkey	Scientist	0.49278875	0.45577568	1	0.60770091
Turkey	Self	-0.1009275	0.87902627	1	0.87902627
United States	Family	-1.3717871	0.13543076	0.27086151	0.14893537
United States	Community	-2.145653	0.02933513	0.11734052	0.1119599
United States	Scientist	-2.4216893	0.05597995	0.16793985	0.1119599
United States	Self	-1.3230694	0.14893537	0.27086151	0.14893537

Table S5. Difference in Positive Reactions w/ Placebo

Country	Estimate	Std.error	Statistic	P-value
United States	-0.000321	0.08070268	-0.0039774	0.99682652
Germany	-0.0827703	0.08066036	-1.026158	0.30481714
Scientist	-0.0520873	0.0808059	-0.6445974	0.51918816
Risk	-0.1259861	0.06996919	-1.8005939	0.07176693
Norms	-0.1065714	0.08074044	-1.3199254	0.18685995
Family	-0.0008626	0.08040474	-0.0107285	0.9914401
Community	-0.1151873	0.08048345	-1.4311919	0.15237526
Self	0.10166216	0.08054322	1.26220633	0.20687457

Table S6. Difference in Negative Reactions w/ Placebo

Country	Estimate	Std.error	Statistic	P-value
United States	-0.0066772	0.01469456	-0.4543968	0.64954328
Germany	0.02685102	0.01468682	1.82824008	0.06751355
Scientist	-0.0064987	0.01471334	-0.4416907	0.65871302
Risk	0.01027959	0.01274015	0.80686579	0.41974382
Norms	0.03369515	0.01470138	2.29197131	0.02190731
Family	0.01327035	0.0146403	0.90642581	0.36471053
Community	0.01333444	0.01465459	0.90991506	0.36286732
Self	-0.00003961	0.01466556	-0.0027011	0.99784485

Draft

2. Permutation-based Inference

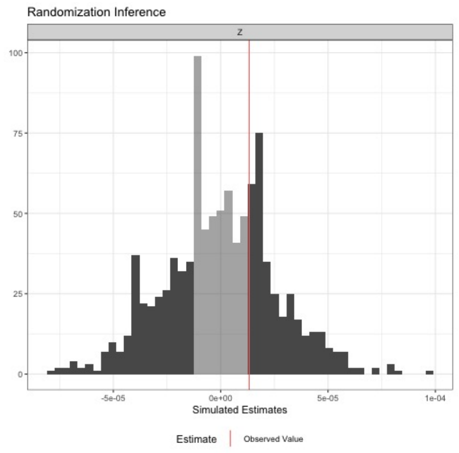

Figure S3. Brazil: US Treatment

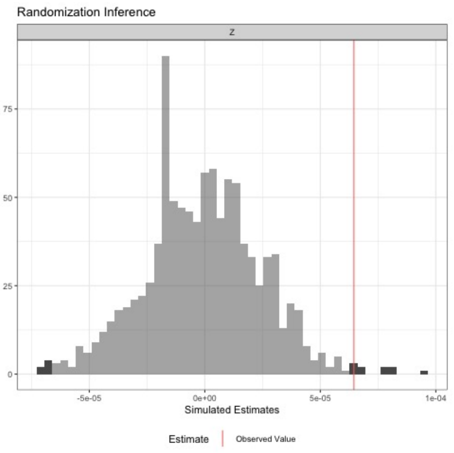

Figure S4. Brazil: Germany Treatment

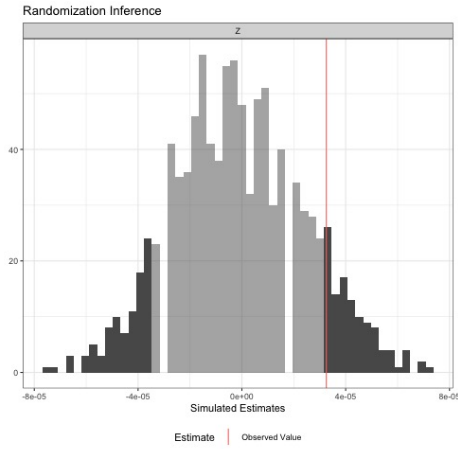

Figure S5. Brazil: Norms Treatment

Draft

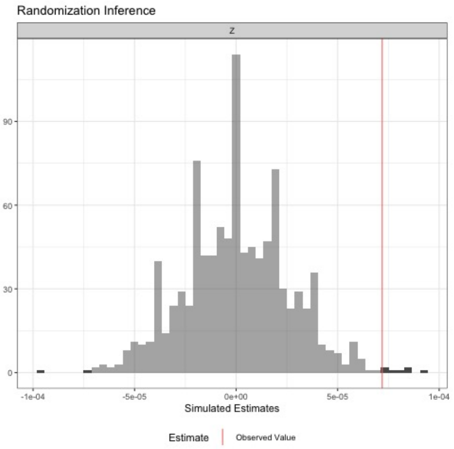

Figure S6. Brazil: Risk Treatment

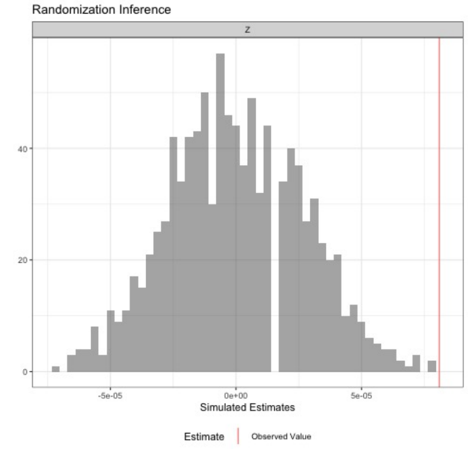

Figure S7. Brazil: Family Treatment

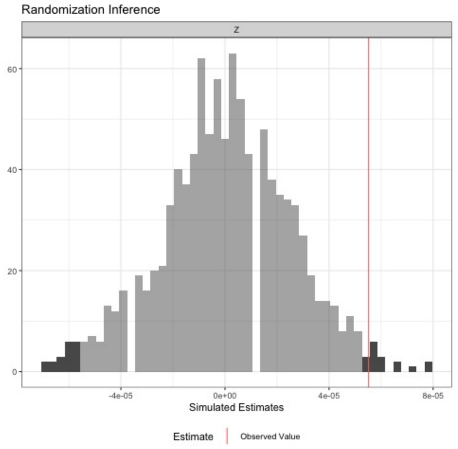

Figure S8. Brazil: Community Treatment

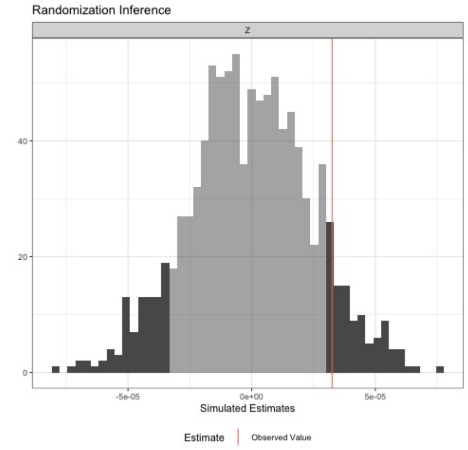

Figure S9. Brazil: Scientist Treatment

Draft

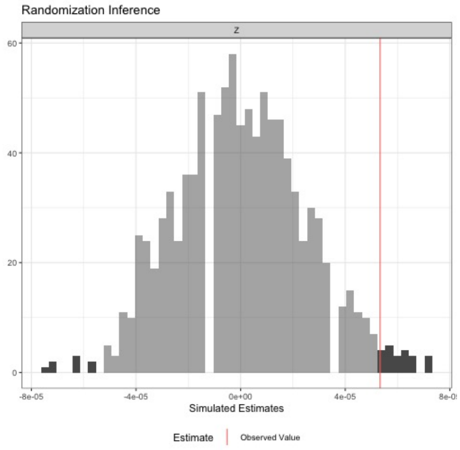

Figure S10. Brazil: Self Treatment

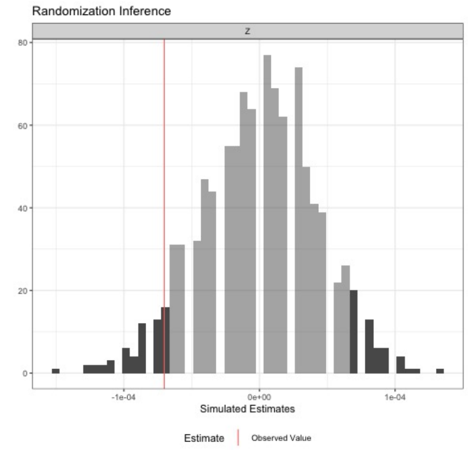

Figure S11. Russia: US Treatment

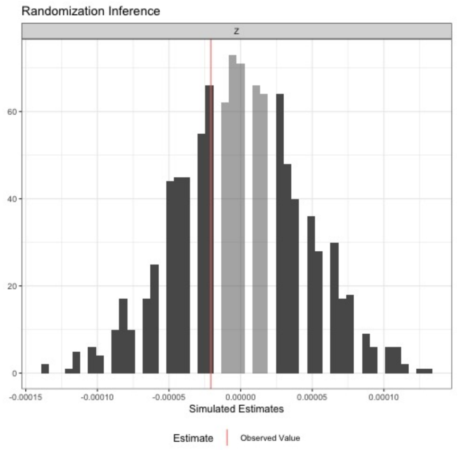

Figure S12. Russia: Germany Treatment

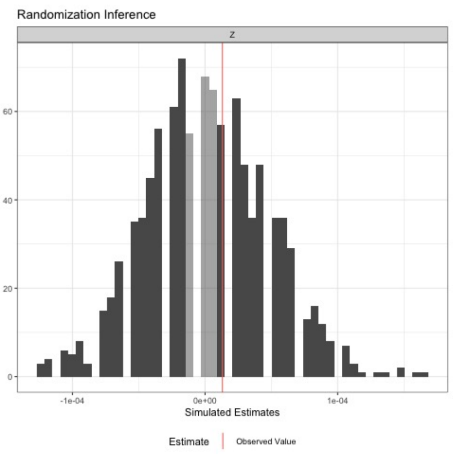

Figure S13. Russia: Norms Treatment

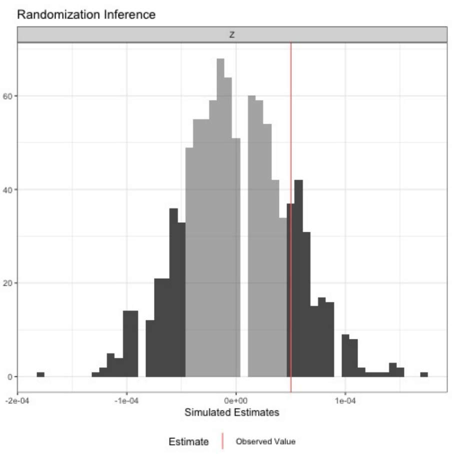

Figure S14. Russia: Risk Treatment

Draft

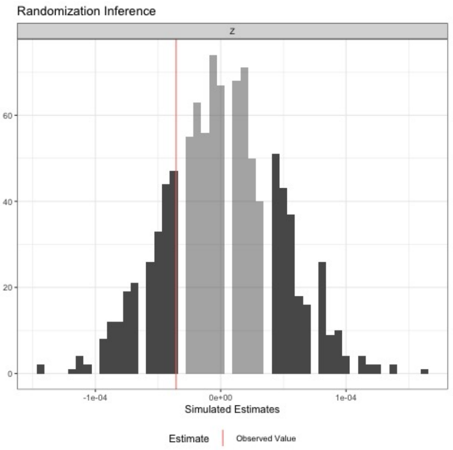

Figure S15. Russia: Family Treatment

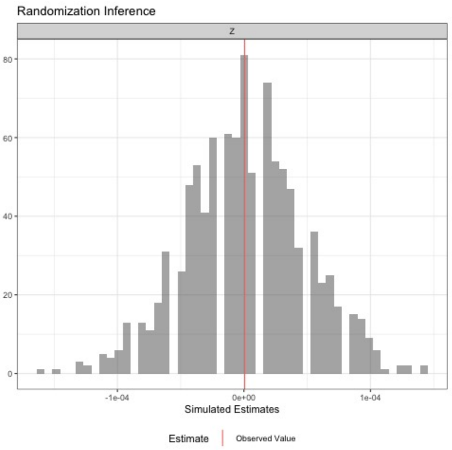

Figure S16. Russia: Community Treatment

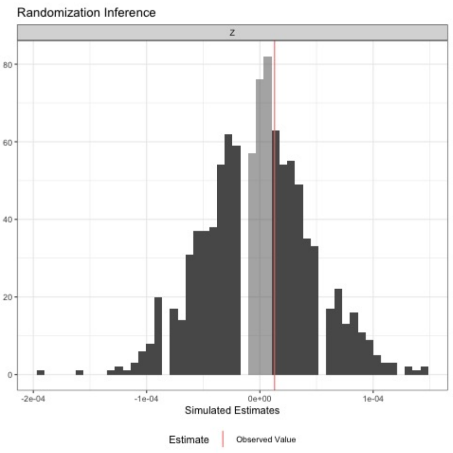

Figure S17. Russia: Scientist Treatment

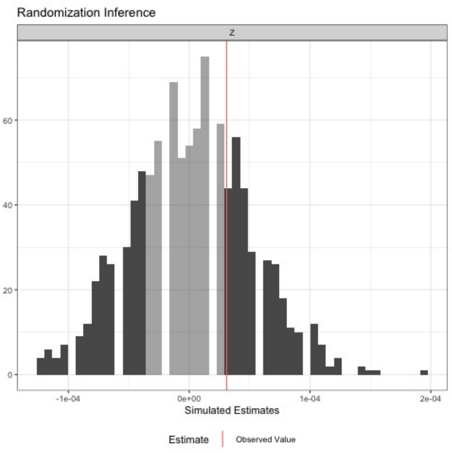

Figure S18. Russia: Self Treatment

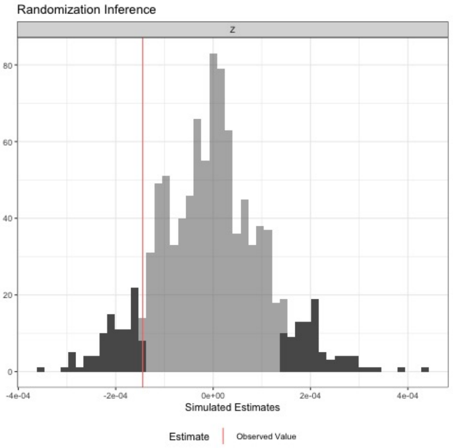

Figure S19. South Africa: US Treatment

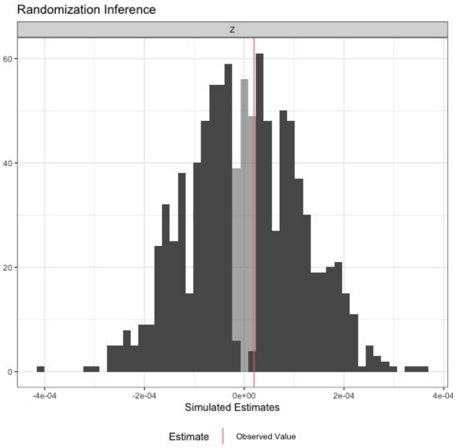

Figure S20. South Africa: Germany Treatment

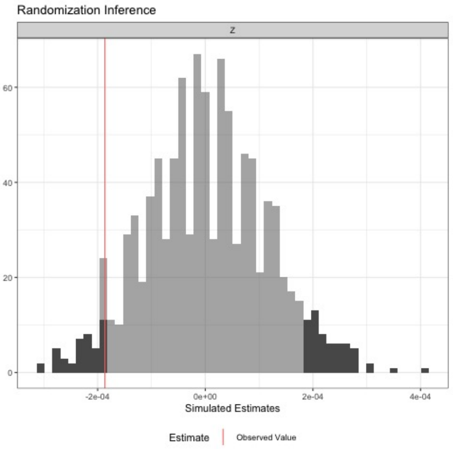

Figure S21. South Africa: Norms Treatment

Draft

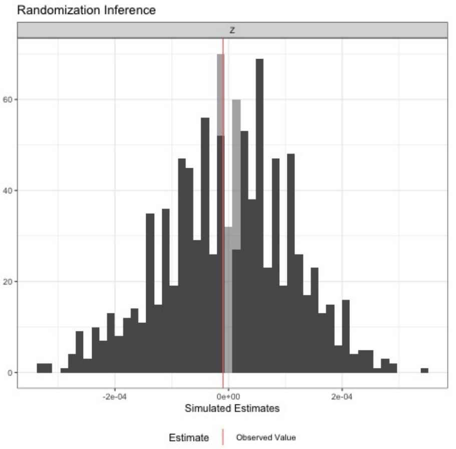

Figure S22. South Africa: Risk Treatment

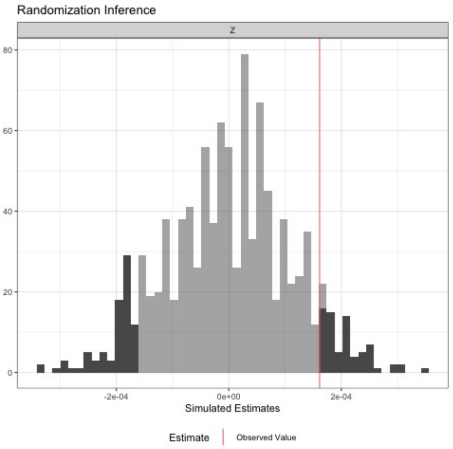

Figure S23. South Africa: Family Treatment

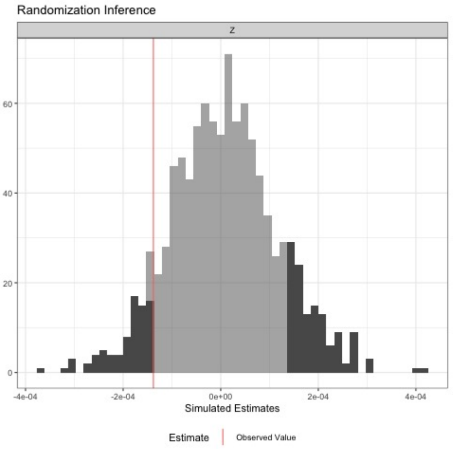

Figure S24. South Africa: Community Treatment

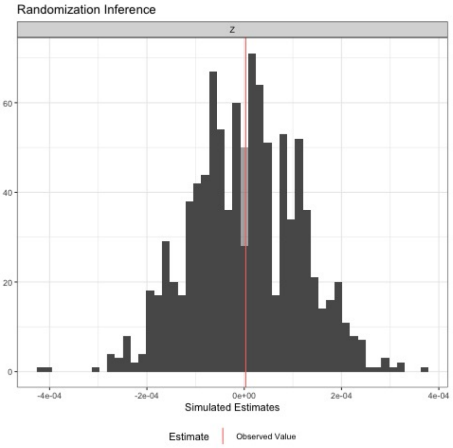

Figure S25. South Africa: Scientist Treatment

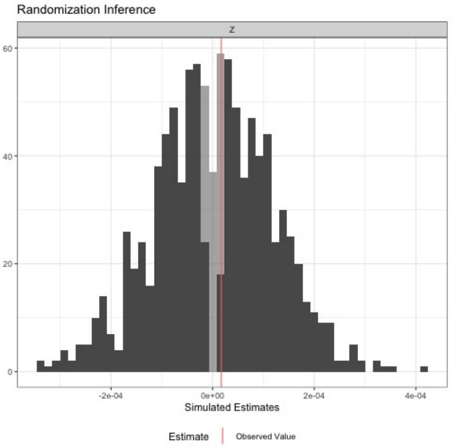

Figure S26. South Africa: Self Treatment

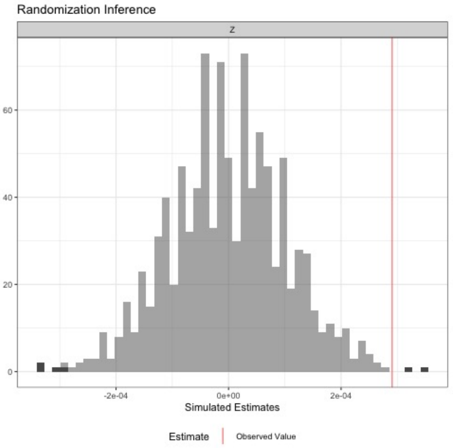

Figure S27. Taiwan: US Treatment

Draft

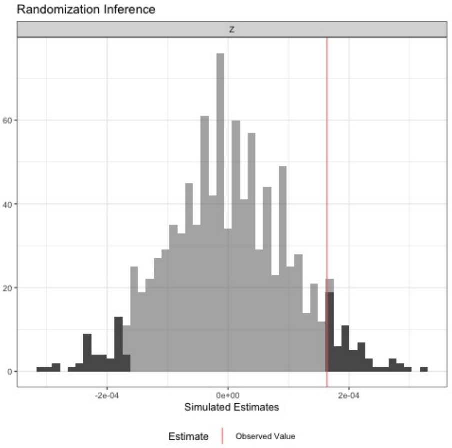

Figure S28. Taiwan: Germany Treatment

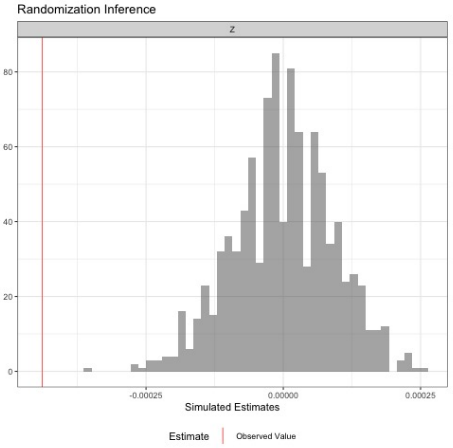

Figure S29. Taiwan: Norms Treatment

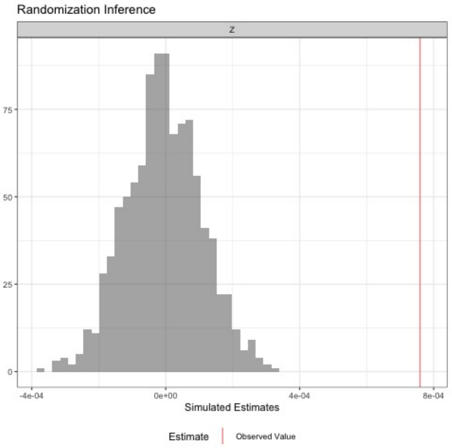

Figure S30. Taiwan: Risk Treatment

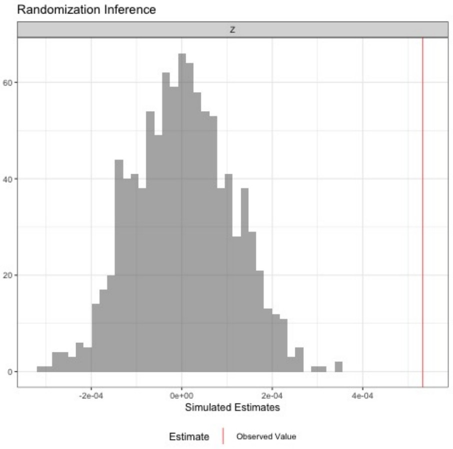

Figure S31. Taiwan: Family Treatment

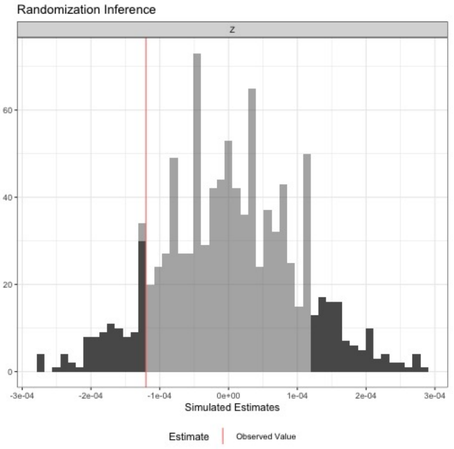

Figure S32. Taiwan: Community Treatment

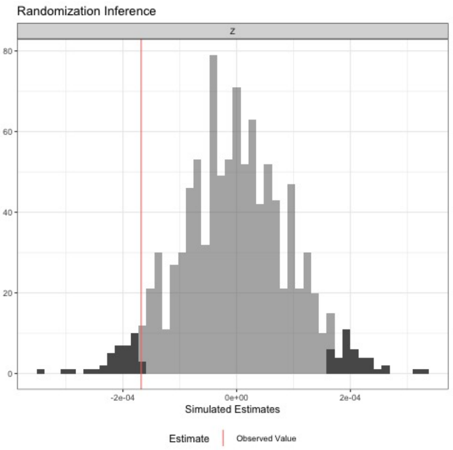

Figure S33. Taiwan: Scientist Treatment

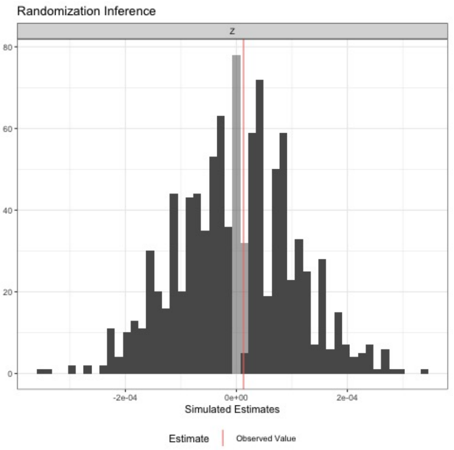

Figure S34. Taiwan: Self Treatment

Draft

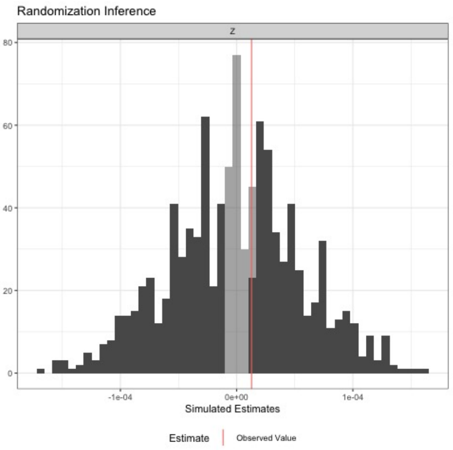

Figure S35. Turkey: US Treatment

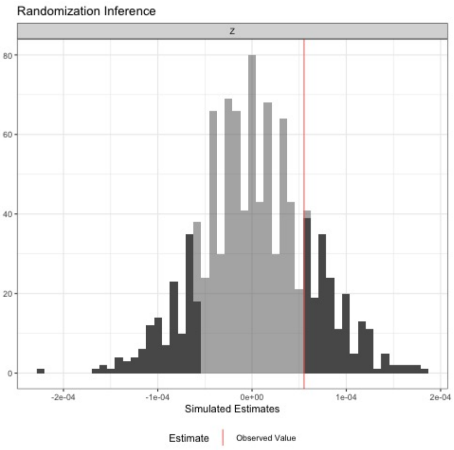

Figure S36. Turkey: Germany Treatment

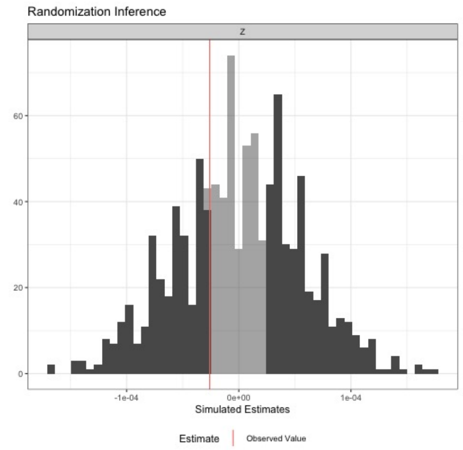

Figure S37. Turkey: Norms Treatment

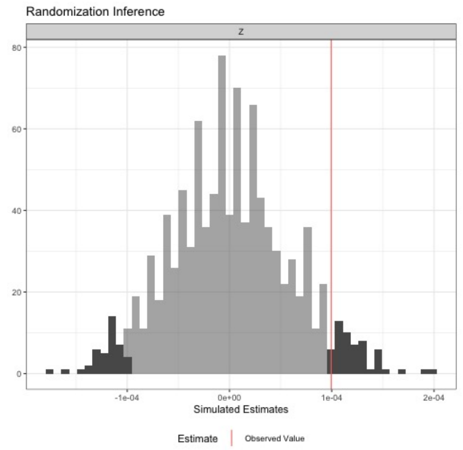

Figure S38. Turkey: Risk Treatment

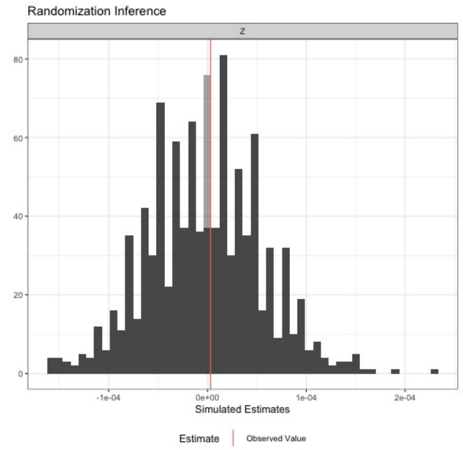

Figure S39. Turkey: Family Treatment

Draft

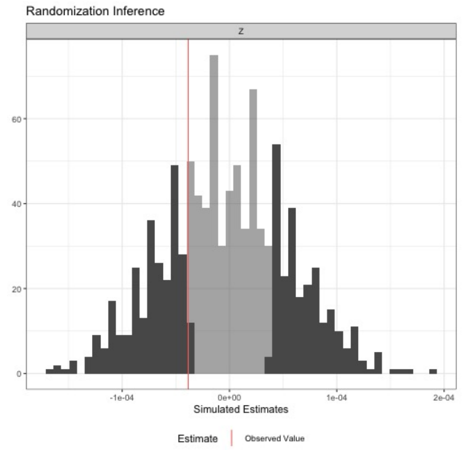

Figure S40. Turkey: Community Treatment

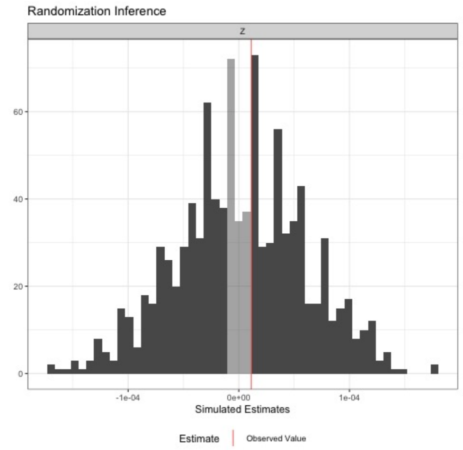

Figure S41. Turkey: Scientist Treatment

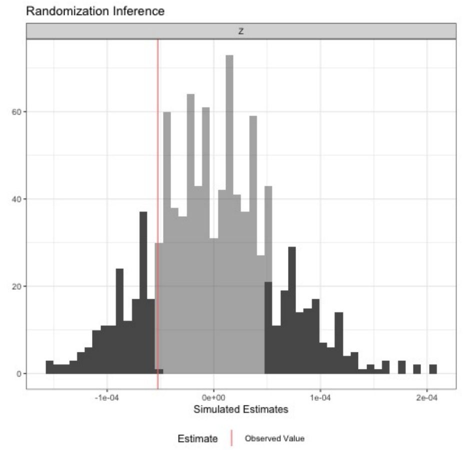

Figure S42. Turkey: Self Treatment

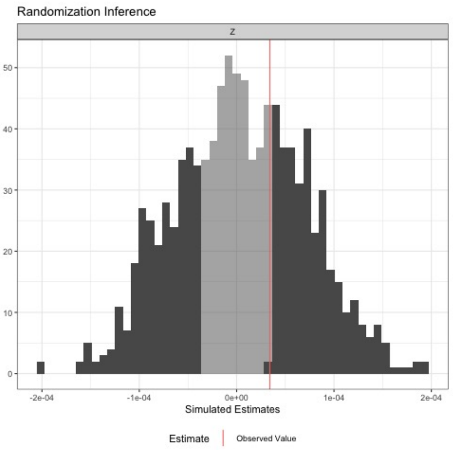

Figure S43. US: US Treatment

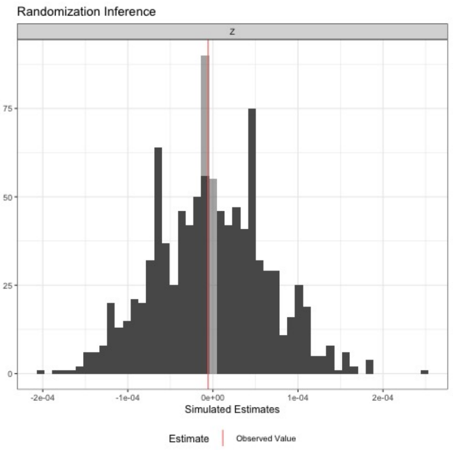

Figure S44. US: Germany Treatment

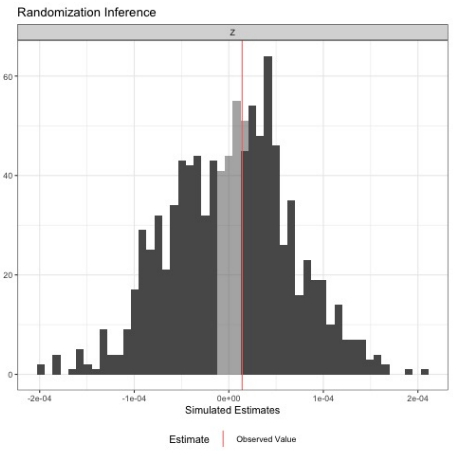

Figure S45. US: Norms Treatment

Draft

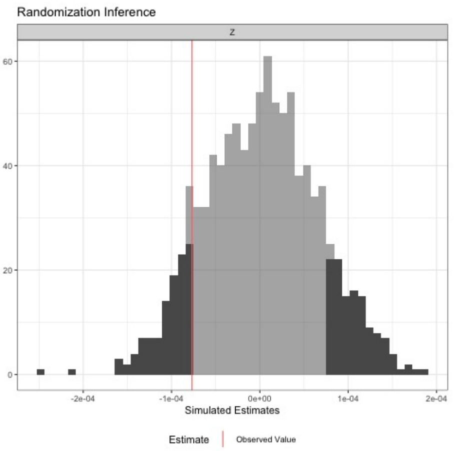

Figure S46. US: Risk Treatment

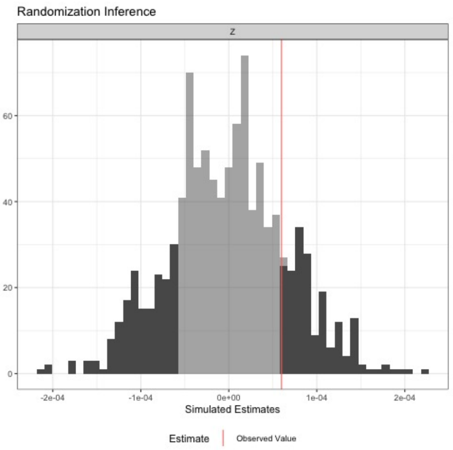

Figure S47. US: Family Treatment

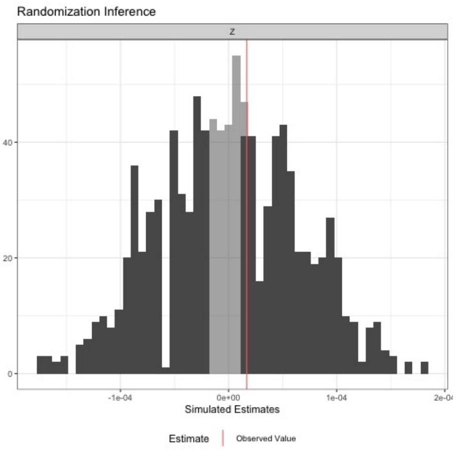

Figure S48. US: Community Treatment

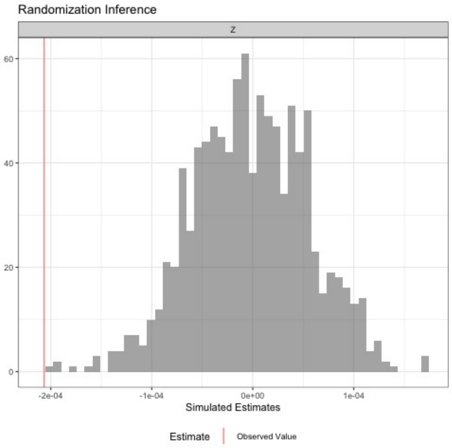

Figure S49. US: Scientist Treatment

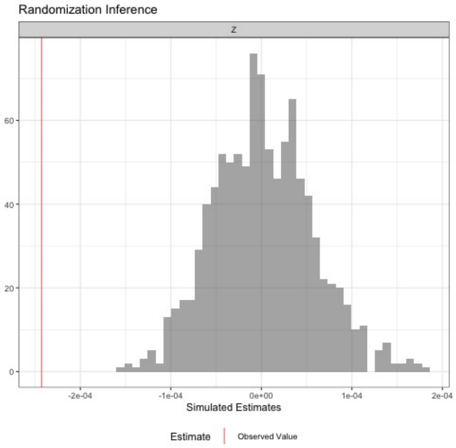

Figure S50. US: Self Treatment

Draft

3. Experiment Details

Table S7. Link Clicks and Reach by Treatment/Country: Brazil (São Paulo), Russia, and South Africa

	Brazil (São Paulo)		Russia		South Africa	
	Link Clicks	Reach	Link Clicks	Reach	Link Clicks	Reach
Placebo1	95	467094	54	195330	264	170432
Placebo2	82	471936	62	196676	235	173196
Family	127	471293	51	195933	281	174100
Self	114	471350	63	192700	250	170228
Community	114	467781	59	199002	229	174255
Scientists	105	475037	60	194233	248	170449
US	94	465869	46	203878	223	170620
Germany	119	470278	54	196277	256	173970
Norms	103	465959	61	197425	218	172241
Risk	122	468378	66	190650	245	169913
Total	1075	4694975	576	1962104	2449	1719404

Table S8. Link Clicks and Reach by Treatment/Country: Taiwan, Turkey, and the United States

	Taiwan		Turkey		United States	
	Link Clicks	Reach	Link Clicks	Reach	Link Clicks	Reach
Placebo1	199	172606	165	279689	112	190237
Placebo2	191	175533	189	284474	92	190832
Family	289	173903	178	282334	115	193607
Self	196	172997	160	278311	61	217870
Community	167	167147	169	287333	105	189949
Scientists	164	172155	184	287871	67	207965
US	245	173408	179	280247	108	189560
Germany	225	174963	198	290046	100	188779
Norms	118	173582	172	286504	106	192668
Risk	350	185473	207	284893	93	214671
Total	2144	1741767	1801	2841702	959	1976138

Draft

Table S9. Brazil (São Paulo): A/B Test Results by Round and Treatment

Round	Start	End	Treatment	Reach	Link Clicks	Click per 10,000 Reach
1	Dec. 4, 2021	Dec. 7, 2021	Placebo	206577	29	1.404
1	Dec. 4, 2021	Dec. 7, 2021	Family	105427	23	2.182
1	Dec. 4, 2021	Dec. 7, 2021	Self	104937	29	2.764
1	Dec. 4, 2021	Dec. 7, 2021	Community	105609	15	1.420
1	Dec. 4, 2021	Dec. 7, 2021	Scientists	106183	22	2.072
1	Dec. 4, 2021	Dec. 7, 2021	US	104365	18	1.725
1	Dec. 4, 2021	Dec. 7, 2021	Germany	102913	29	2.818
1	Dec. 4, 2021	Dec. 7, 2021	Norms	102782	23	2.238
1	Dec. 4, 2021	Dec. 7, 2021	Risk	103319	31	3.000
Round 1				1042112	219	2.102
2	Dec. 10, 2021	Dec. 14, 2021	Placebo	376657	78	2.071
2	Dec. 10, 2021	Dec. 14, 2021	Family	190125	55	2.893
2	Dec. 10, 2021	Dec. 14, 2021	Self	188773	47	2.490
2	Dec. 10, 2021	Dec. 14, 2021	Community	187191	56	2.992
2	Dec. 10, 2021	Dec. 14, 2021	Scientists	190348	53	2.784
2	Dec. 10, 2021	Dec. 14, 2021	US	187571	40	2.133
2	Dec. 10, 2021	Dec. 14, 2021	Germany	189114	54	2.855
2	Dec. 10, 2021	Dec. 14, 2021	Norms	188998	40	2.116
2	Dec. 10, 2021	Dec. 14, 2021	Risk	186760	44	2.356
Round 2				1885537	467	2.477
3	Dec. 18, 2021	Dec. 21, 2021	Placebo	355796	70	1.967
3	Dec. 18, 2021	Dec. 21, 2021	Family	175741	49	2.788
3	Dec. 18, 2021	Dec. 21, 2021	Self	177640	38	2.139
3	Dec. 18, 2021	Dec. 21, 2021	Community	174981	43	2.457
3	Dec. 18, 2021	Dec. 21, 2021	Scientists	178506	30	1.681
3	Dec. 18, 2021	Dec. 21, 2021	US	173933	36	2.071
3	Dec. 18, 2021	Dec. 21, 2021	Germany	178251	36	2.020
3	Dec. 18, 2021	Dec. 21, 2021	Norms	174179	40	2.296
3	Dec. 18, 2021	Dec. 21, 2021	Risk	178299	47	2.636
Round 3				1767326	389	2.201
Total				4694975	1075	2.290

Draft

Table S10. Russia: A/B Test Results by Round and Treatment

Round	Start	End	Treatment	Reach	Link Clicks	Click per 10,000 Reach
1	Dec. 3, 2021	Dec. 6, 2021	Placebo	84585	28	3.310
1	Dec. 3, 2021	Dec. 6, 2021	Family	42144	5	1.186
1	Dec. 3, 2021	Dec. 6, 2021	Self	42006	13	3.095
1	Dec. 3, 2021	Dec. 6, 2021	Community	40631	11	2.707
1	Dec. 3, 2021	Dec. 6, 2021	Specialists	41317	13	3.146
1	Dec. 3, 2021	Dec. 6, 2021	US	43188	9	2.084
1	Dec. 3, 2021	Dec. 6, 2021	Germany	41648	7	1.681
1	Dec. 3, 2021	Dec. 6, 2021	Norms	42299	11	2.610
1	Dec. 3, 2021	Dec. 6, 2021	Risk	40027	13	3.248
Round 1				417845	110	2.633
2	Dec. 10, 2021	Dec. 14, 2021	Placebo	307427	88	2.863
2	Dec. 10, 2021	Dec. 14, 2021	Family	153789	46	2.991
2	Dec. 10, 2021	Dec. 14, 2021	Self	150694	50	3.318
2	Dec. 10, 2021	Dec. 14, 2021	Community	158371	48	3.031
2	Dec. 10, 2021	Dec. 14, 2021	Specialists	152916	47	3.074
2	Dec. 10, 2021	Dec. 14, 2021	US	160690	37	2.303
2	Dec. 10, 2021	Dec. 14, 2021	Germany	154629	47	3.040
2	Dec. 10, 2021	Dec. 14, 2021	Norms	155126	51	3.223
2	Dec. 10, 2021	Dec. 14, 2021	Risk	150623	53	3.519
Round 2				1544259	466	3.018
Total				1962104	576	2.936

Table S11. South Africa: A/B Test Results by Round and Treatment

Round	Start	End	Treatment	Reach	Link Clicks	Click per 10,000 Reach
1	Nov. 4, 2021	Nov. 6, 2021	Placebo	52404	79	15.075
1	Nov. 4, 2021	Nov. 6, 2021	Family	25893	41	15.834
1	Nov. 4, 2021	Nov. 6, 2021	Self	25644	27	10.529
1	Nov. 4, 2021	Nov. 6, 2021	Community	26299	26	9.886
1	Nov. 4, 2021	Nov. 6, 2021	Scientists	26295	27	10.268
1	Nov. 4, 2021	Nov. 6, 2021	US	26382	35	13.267
1	Nov. 4, 2021	Nov. 6, 2021	Germany	26312	48	18.243
1	Nov. 4, 2021	Nov. 6, 2021	Norms	26194	26	9.926
1	Nov. 4, 2021	Nov. 6, 2021	Risk	25822	33	12.780
Round 1				264787	342	13.091
2	Nov. 18, 2021	Nov. 21, 2021	Placebo	100290	141	14.059
2	Nov. 18, 2021	Nov. 21, 2021	Family	51268	80	15.604
2	Nov. 18, 2021	Nov. 21, 2021	Self	50874	70	13.759
2	Nov. 18, 2021	Nov. 21, 2021	Community	51504	72	13.979
2	Nov. 18, 2021	Nov. 21, 2021	Scientists	49784	82	16.471
2	Nov. 18, 2021	Nov. 21, 2021	US	51089	67	13.114
2	Nov. 18, 2021	Nov. 21, 2021	Germany	50792	73	14.372
2	Nov. 18, 2021	Nov. 21, 2021	Norms	50566	83	16.414
2	Nov. 18, 2021	Nov. 21, 2021	Risk	50586	70	13.838
Round 2				506753	738	14.563
3	Dec. 6, 2021	Dec. 10, 2021	Placebo	190934	279	14.612
3	Dec. 6, 2021	Dec. 10, 2021	Family	96939	160	16.505
3	Dec. 6, 2021	Dec. 10, 2021	Self	93710	153	16.327
3	Dec. 6, 2021	Dec. 10, 2021	Community	96452	131	13.582
3	Dec. 6, 2021	Dec. 10, 2021	Scientists	94370	139	14.729
3	Dec. 6, 2021	Dec. 10, 2021	US	93149	121	12.099
3	Dec. 6, 2021	Dec. 10, 2021	Germany	96866	135	13.937
3	Dec. 6, 2021	Dec. 10, 2021	Norms	95481	109	11.416
3	Dec. 6, 2021	Dec. 10, 2021	Risk	93505	142	15.186
Round 3				951406	1369	14.389
Total				1719404	2449	14.243

Draft

Table S12. Taiwan: A/B Test Results by Round and Treatment

Round	Start	End	Treatment	Reach	Link Clicks	Click per 10,000 Reach
1	Nov. 1, 2021	Nov. 3, 2021	Placebo	131295	150	11.425
1	Nov. 1, 2021	Nov. 4, 2021	Family	62342	79	12.672
1	Nov. 1, 2021	Nov. 4, 2021	Self	65071	84	12.909
1	Nov. 1, 2021	Nov. 4, 2021	Community	62239	71	11.408
1	Nov. 1, 2021	Nov. 4, 2021	Scientists	63476	55	8.665
1	Nov. 1, 2021	Nov. 3, 2021	US	66338	105	15.828
1	Nov. 1, 2021	Nov. 3, 2021	Germany	67209	100	14.879
1	Nov. 1, 2021	Nov. 3, 2021	Norms	66340	38	5.728
1	Nov. 1, 2021	Nov. 3, 2021	Risk	67476	102	15.116
Round 1				651786	784	12.028
2	Nov. 18, 2021	Nov. 24, 2021	Placebo	216844	240	11.068
2	Nov. 18, 2021	Nov. 24, 2021	Family	111561	210	18.824
2	Nov. 18, 2021	Nov. 24, 2021	Self	107926	112	10.377
2	Nov. 18, 2021	Nov. 24, 2021	Community	104908	96	9.151
2	Nov. 18, 2021	Nov. 24, 2021	Scientists	108679	109	10.030
2	Nov. 18, 2021	Nov. 24, 2021	US	107070	140	13.076
2	Nov. 18, 2021	Nov. 24, 2021	Germany	107754	125	11.600
2	Nov. 18, 2021	Nov. 24, 2021	Norms	107242	80	7.460
2	Nov. 18, 2021	Nov. 24, 2021	Risk	117997	248	21.017
Round 2				1089981	1360	12.477
Total				1741767	2144	12.309

Table S13. Turkey: A/B Test Results by Round and Treatment

Round	Start	End	Treatment	Reach	Link Clicks	Click per 10,000 Reach
1	Oct. 26, 2021	Nov. 4, 2021	Placebo	313717	215	6.853
1	Oct. 26, 2021	Nov. 4, 2021	Family	156157	98	6.276
1	Oct. 26, 2021	Nov. 4, 2021	Self	154417	84	5.440
1	Oct. 26, 2021	Nov. 4, 2021	Community	156698	96	6.126
1	Oct. 26, 2021	Nov. 4, 2021	Scientists	157944	89	5.635
1	Oct. 26, 2021	Nov. 4, 2021	US	152339	106	6.958
1	Oct. 26, 2021	Nov. 4, 2021	Germany	160828	111	6.902
1	Oct. 26, 2021	Nov. 4, 2021	Norms	157350	107	6.800
1	Oct. 26, 2021	Nov. 4, 2021	Risk	156065	114	7.305
Round 1				1565515	1020	6.515
2	Nov. 6, 2021	Nov. 9, 2021	Placebo	250446	139	5.550
2	Nov. 6, 2021	Nov. 9, 2021	Family	126177	80	6.340
2	Nov. 6, 2021	Nov. 9, 2021	Self	123894	76	6.134
2	Nov. 6, 2021	Nov. 9, 2021	Community	130635	73	5.588
2	Nov. 6, 2021	Nov. 9, 2021	Scientists	129927	95	7.312
2	Nov. 6, 2021	Nov. 9, 2021	US	127908	73	5.707
2	Nov. 6, 2021	Nov. 9, 2021	Germany	129218	87	6.733
2	Nov. 6, 2021	Nov. 9, 2021	Norms	129154	65	5.033
2	Nov. 6, 2021	Nov. 9, 2021	Risk	128828	93	7.219
Round 2				1276187	781	6.120
Total				2841702	1801	6.338

Table S14. United States: A/B Test Results by Round and Treatment

Draft

Round	Start	End	Treatment	Reach	Link Clicks	Click per 10,000 Reach
1	Oct. 14, 2021	Oct. 17, 2021	Placebo	112131	58	5.173
1	Oct. 14, 2021	Oct. 17, 2021	Family	56433	23	4.076
1	Oct. 14, 2021	Oct. 17, 2021	Self	58271	26	4.462
1	Oct. 14, 2021	Oct. 17, 2021	Community	56324	40	7.102
1	Oct. 14, 2021	Oct. 17, 2021	Scientists	55372	28	5.057
1	Oct. 14, 2021	Oct. 17, 2021	US	56325	28	4.971
1	Oct. 14, 2021	Oct. 17, 2021	Germany	54606	23	4.212
1	Oct. 14, 2021	Oct. 17, 2021	Norms	55682	26	4.669
1	Oct. 14, 2021	Oct. 17, 2021	Risk	55080	39	7.081
Round 1				560224	291	5.194
2	Dec. 7, 2021	Dec. 15, 2021	Placebo	79081	66	8.346
2	Dec. 7, 2021	Dec. 15, 2021	Family	39053	32	8.194
2	Dec. 7, 2021	Dec. 15, 2021	Self	40117	15	3.739
2	Dec. 7, 2021	Dec. 15, 2021	Community	39534	25	6.324
2	Dec. 7, 2021	Dec. 15, 2021	Scientists	56928	4	0.703
2	Dec. 7, 2021	Dec. 15, 2021	US	39751	26	6.541
2	Dec. 7, 2021	Dec. 15, 2021	Germany	39166	34	8.681
2	Dec. 7, 2021	Dec. 15, 2021	Norms	40385	37	9.162
2	Dec. 7, 2021	Dec. 15, 2021	Risk	39845	31	7.780
Round 2				413860	270	6.524
3	Jan. 13, 2022	Jan. 18, 2022	Placebo	189857	80	4.214
3	Jan. 13, 2022	Jan. 18, 2022	Family	98121	60	6.115
3	Jan. 13, 2022	Jan. 18, 2022	Self	119482	20	1.674
3	Jan. 13, 2022	Jan. 18, 2022	Community	94091	40	4.251
3	Jan. 13, 2022	Jan. 18, 2022	Scientists	95665	35	3.659
3	Jan. 13, 2022	Jan. 18, 2022	US	93484	56	5.776
3	Jan. 13, 2022	Jan. 18, 2022	Germany	95007	44	4.526
3	Jan. 13, 2022	Jan. 18, 2022	Norms	96601	43	4.451
3	Jan. 13, 2022	Jan. 18, 2022	Risk	119746	23	1.921
Round 3				1002054	398	3.972
Total				1976138	959	4.853

4. Vaccination Rate by Country

Table S15. Vaccination Rate by Country

Country	Date	Vaccinated (%)	Fully Vaccinated (%)
Brazil	Dec. 4, 2021	76.89%	63.94%
Brazil	Dec. 7, 2021	76.97%	64.44%
Brazil	Dec. 10, 2021	77.07%	65.11%
Brazil	Dec. 14, 2021	77.07%	65.49%
Brazil	Dec. 18, 2021	77.30%	66.11%
Brazil	Dec. 21, 2021	77.36%	66.32%
Russia	Dec. 3, 2021	46.24%	39.66%
Russia	Dec. 6, 2021	46.68%	40.30%
Russia	Dec. 10, 2021	47.37%	41.23%
Russia	Dec. 14, 2021	48.33%	42.38%
South Africa	Nov. 4, 2021	26.07%	21.21%
South Africa	Nov. 6, 2021	26.27%	21.32%
South Africa	Nov. 18, 2021	27.56%	22.92%
South Africa	Nov. 21, 2021	27.73%	23.11%
South Africa	Dec. 6, 2021	29.89%	25.02%
South Africa	Dec. 10, 2021	30.55%	25.53%
Taiwan	Nov. 1, 2021	72.46%	33.10%
Taiwan	Nov. 4, 2021	72.94%	35.43%
Taiwan	Nov. 18, 2021	75.38%	44.84%
Taiwan	Nov. 24, 2021	75.91%	49.56%
Turkey	Oct. 26, 2021	64.98%	56.82%
Turkey	Nov. 4, 2021	65.32%	57.63%
Turkey	Nov. 6, 2021	65.39%	57.80%
Turkey	Nov. 9, 2021	65.49%	58.02%
United States	Oct. 14, 2021	65.52%	58.20%
United States	Oct. 17, 2021	65.66%	58.36%
United States	Dec. 7, 2021	70.97%	61.40%
United States	Dec. 15, 2021	71.79%	62.12%
United States	Jan. 13, 2022	74.45%	63.60%
United States	Jan. 18, 2022	74.80%	63.79%

Draft

[1] People vaccinated was calculated using “people vaccinated/population” and Fully vaccinated (%) was calculated using “people fully vaccinated/population”.

[2] As Brazil for Dec. 10, 2021 is missing “Vaccinated (%)”, we interpolate it at 77.07 percent from the other two adjacent numbers.

5. Facebook Ads Example

Country	Group	Treatment	Ad Image	Group	Treatment	Ad Image
Brazil	A	Norms	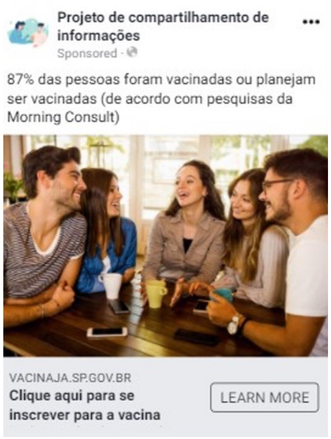	A	Risk	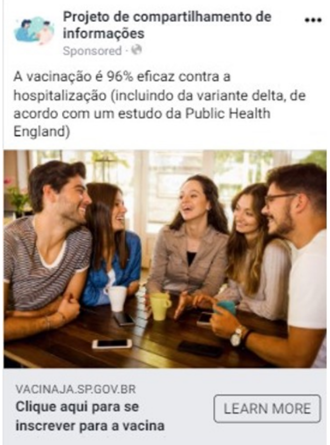
Brazil	A	US	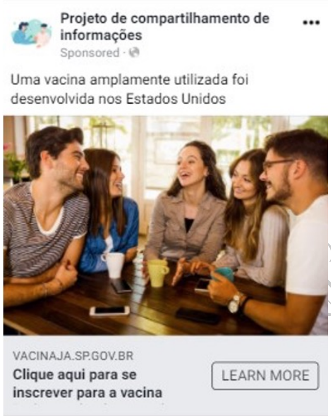	A	Germany	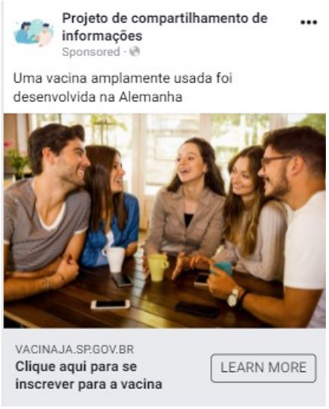
Brazil	A	Placebo	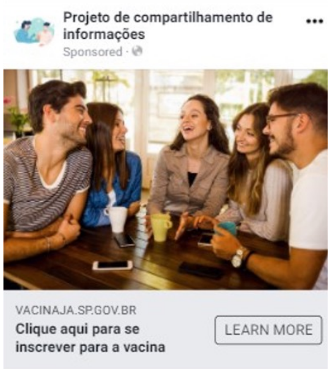			

Table S16. Brazil: Group A Ads

Country	Group	Treatment	Ad Image	Group	Treatment	Ad Image
Brazil	B	Family	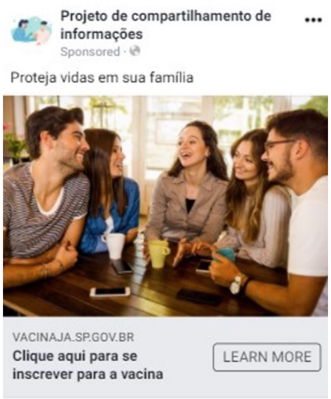	B	Self	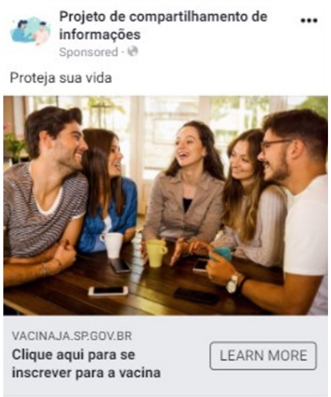
Brazil	B	Community	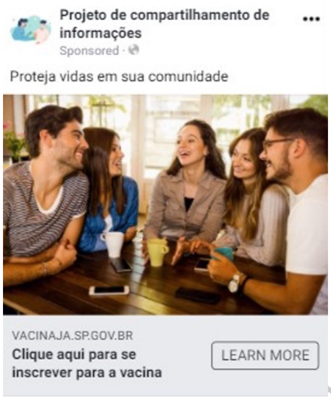	B	Scientist	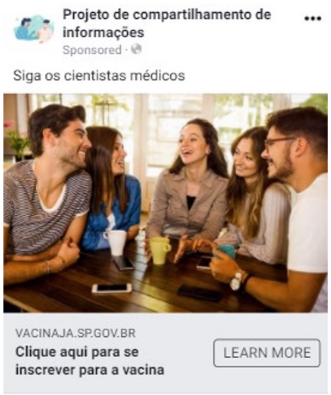
Brazil	B	Placebo	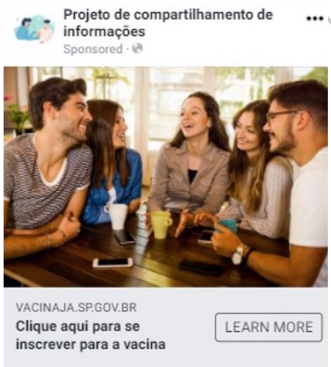			

Table S17. Brazil: Group B Ads

Country	Group	Treatment	Ad Image	Group	Treatment	Ad Image
Russia	A	Norms	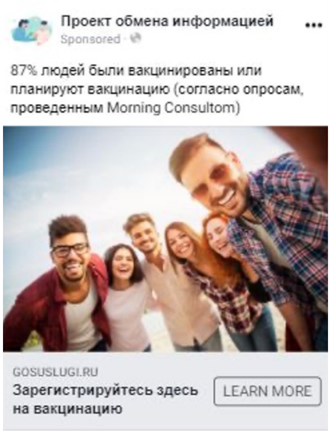	A	Risk	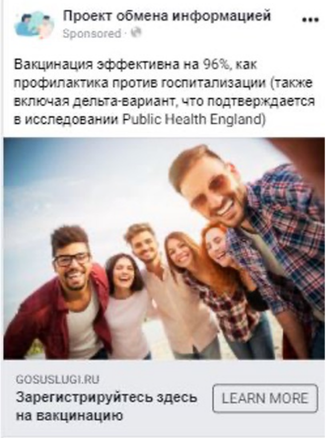
Russia	A	US	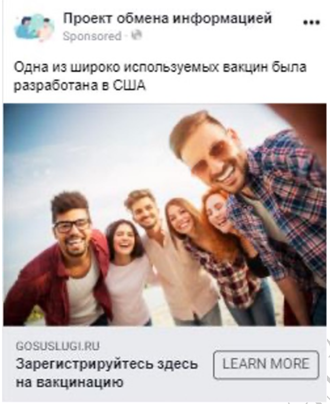	A	Germany	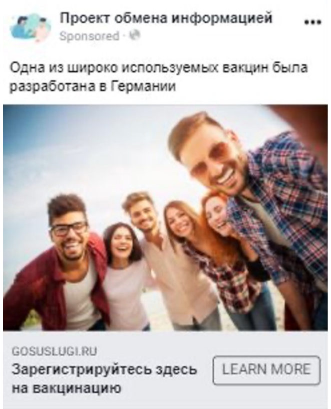
Russia	A	Placebo	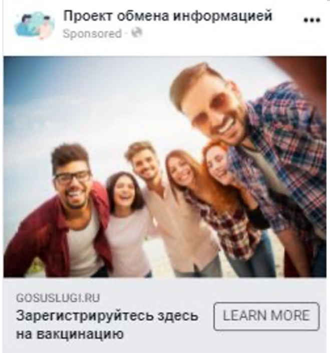			

Table S18. Russia: Group A Ads

Country	Group	Treatment	Ad Image	Group	Treatment	Ad Image
Russia	B	Family	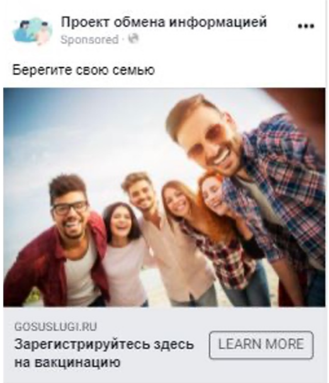	B	Self	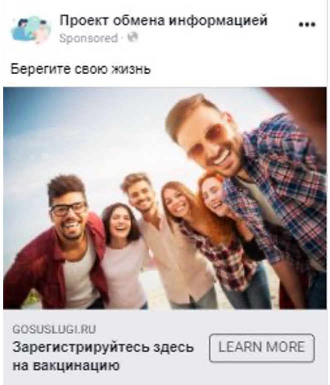
Russia	B	Community	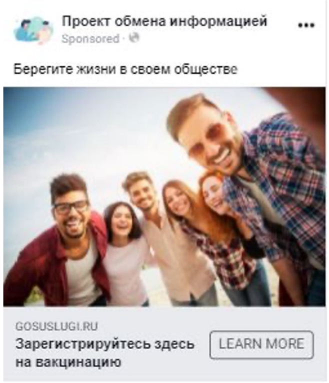	B	Scientist	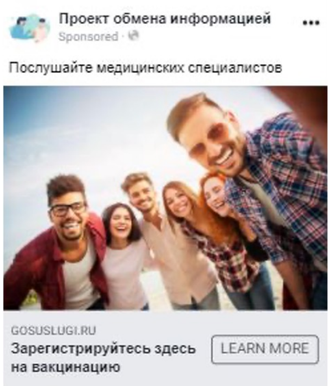
Russia	B	Placebo	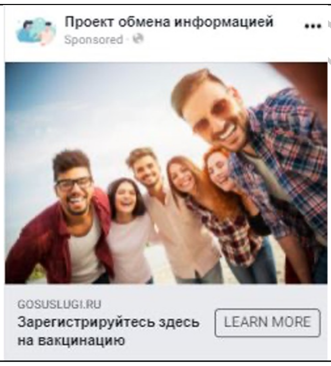			

Table S19. Russia: Group B Ads

Country	Group	Treatment	Ad Image	Group	Treatment	Ad Image
South Africa	A	Norms	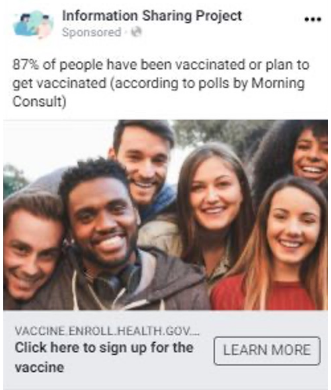	A	Risk	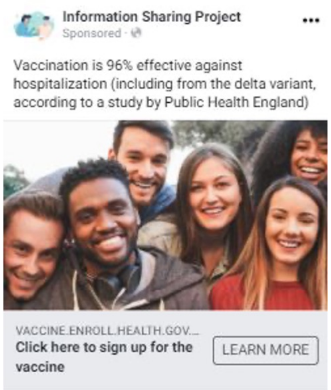
South Africa	A	US	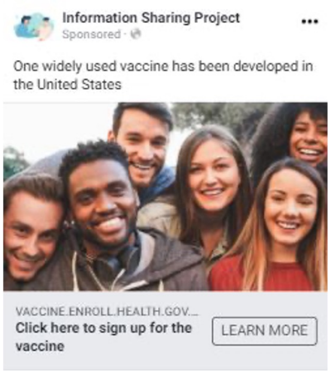	A	Germany	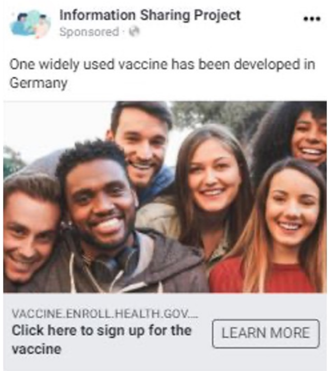
South Africa	A	Placebo	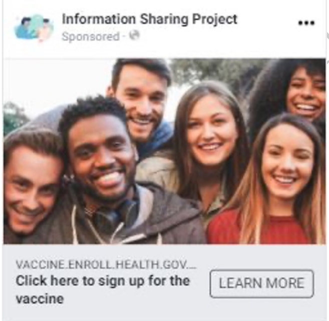			

Table S20. South Africa: Group A Ads

Country	Group	Treatment	Ad Image	Group	Treatment	Ad Image
South Africa	B	Family	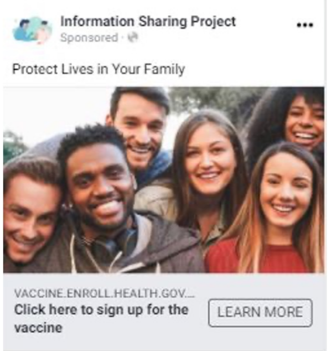	B	Self	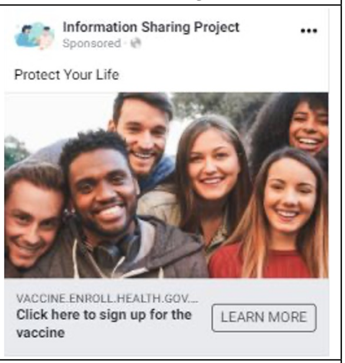
South Africa	B	Community	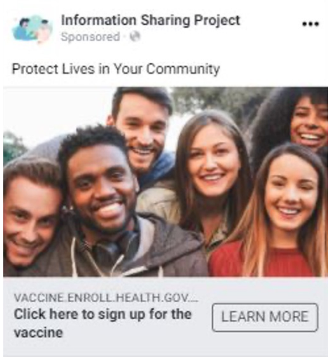	B	Scientist	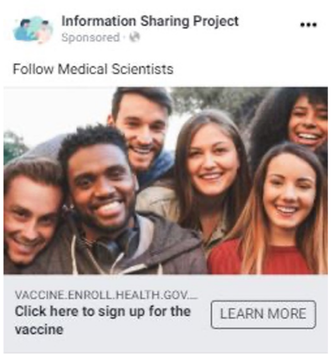
South Africa	B	Placebo	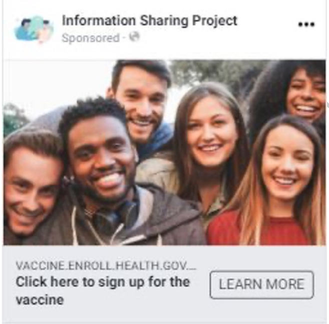			

Table S21. South Africa: Group B Ads

Country	Group	Treatment	Ad Image	Group	Treatment	Ad Image
Taiwan	A	Norms	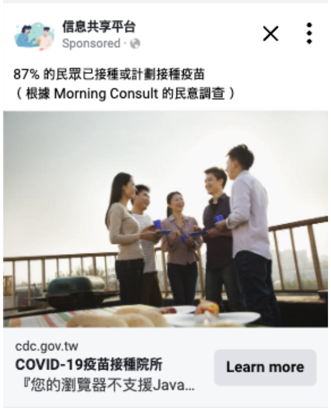	A	Risk	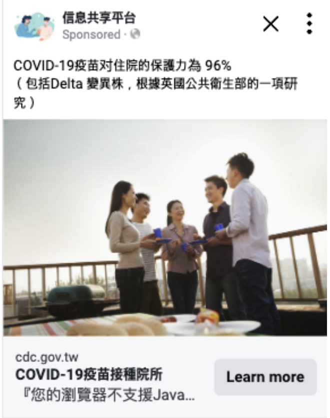
Taiwan	A	US	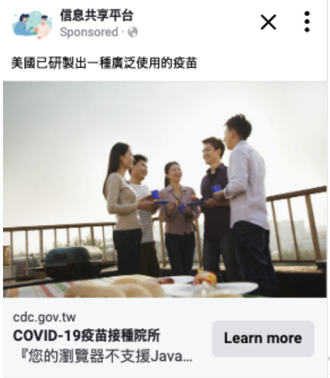	A	Germany	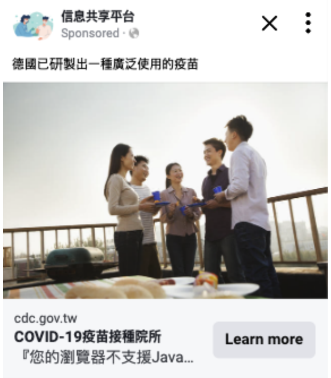
Taiwan	A	Placebo	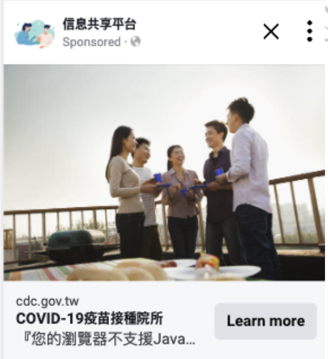			

Table S22. Taiwan: Group A Ads

Country	Group	Treatment	Ad Image	Group	Treatment	Ad Image
Taiwan	B	Family	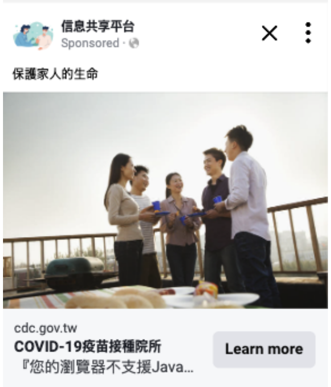	B	Self	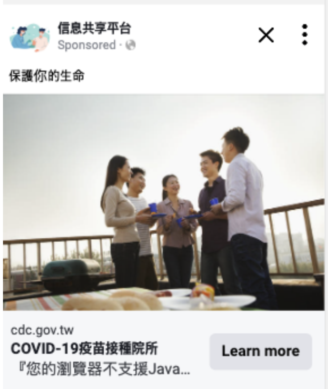
Taiwan	B	Community	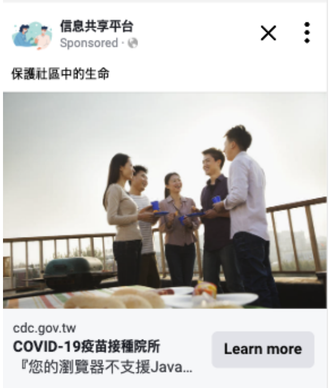	B	Scientist	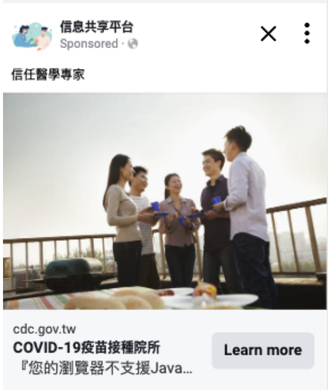
Taiwan	B	Placebo	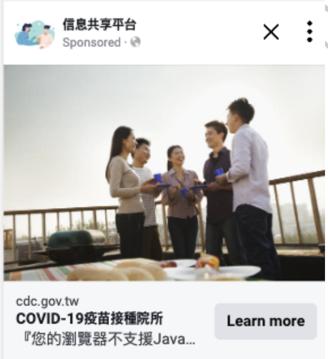			

Table S23. Taiwan: Group B Ads

Country	Group	Treatment	Ad Image	Group	Treatment	Ad Image
Turkey	A	Norms	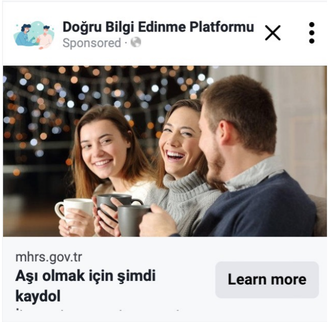	A	Risk	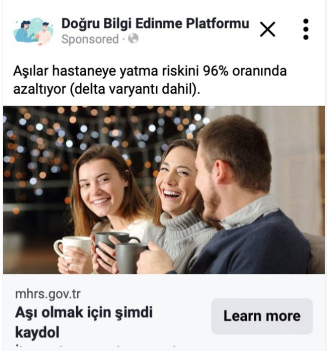
Turkey	A	US	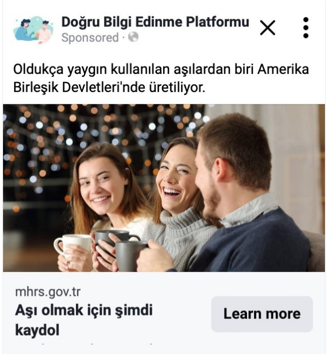	A	Germany	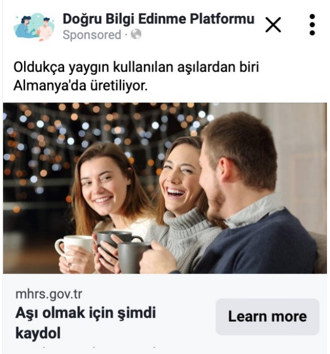
Turkey	A	Placebo	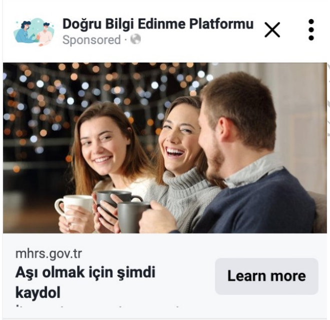			

Table S24. Turkey: Group A Ads

Country	Group	Treatment	Ad Image	Group	Treatment	Ad Image
Turkey	B	Family	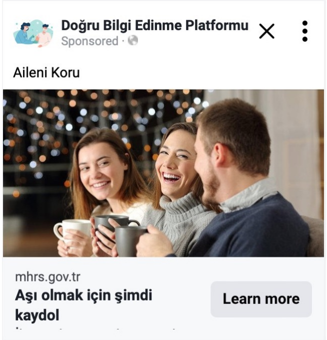	B	Self	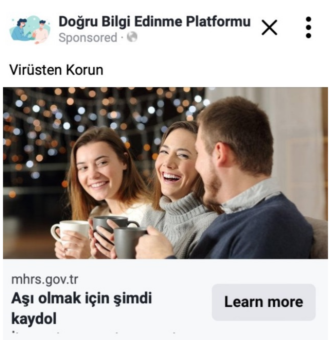
Turkey	B	Community	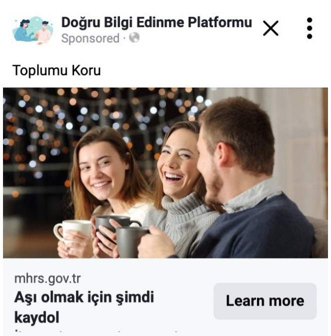	B	Scientist	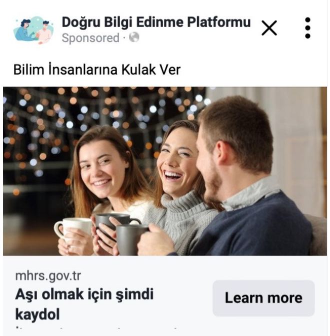
Turkey	B	Placebo	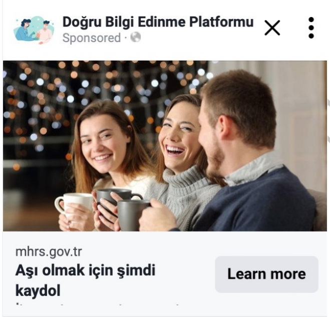			

Table S25. Turkey: Group B Ads

Country	Group	Treatment	Ad Image	Group	Treatment	Ad Image
US	A	Norms	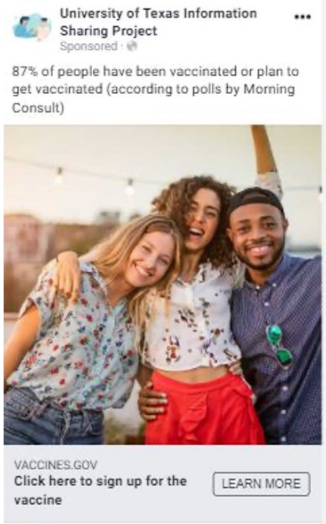	A	Risk	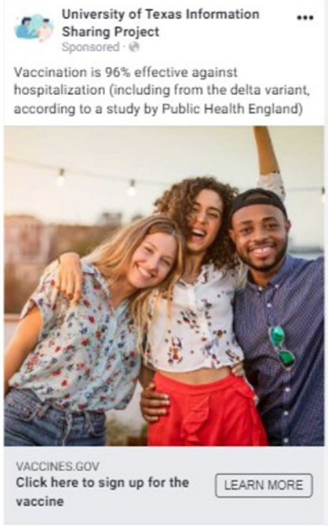
US	A	US	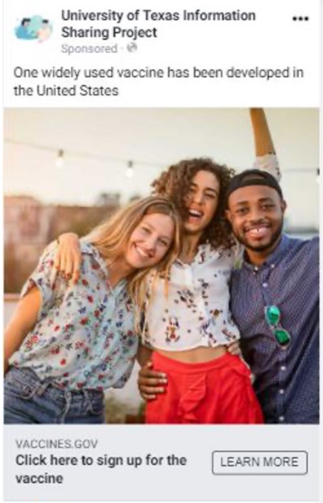	A	Germany	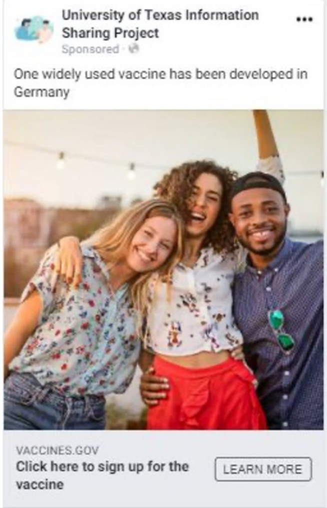
US	A	Placebo	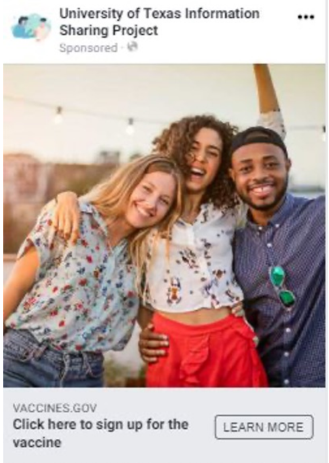			

Table S26. US: Group A Ads

<table>
<tr><th>Country</th><th>Group</th><th>Treatment</th><th>Ad Image</th><th>Group</th><th>Treatment</th><th>Ad Image</th></tr>
<tr><td>US</td><td>B</td><td>Family</td><td>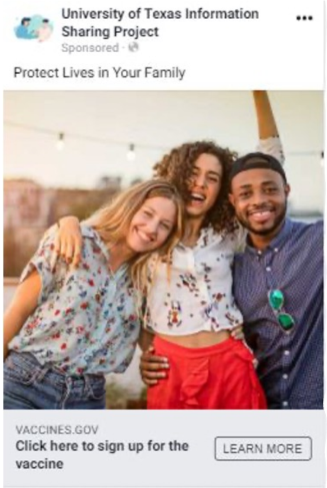
</td><td>B</td><td>Self</td><td>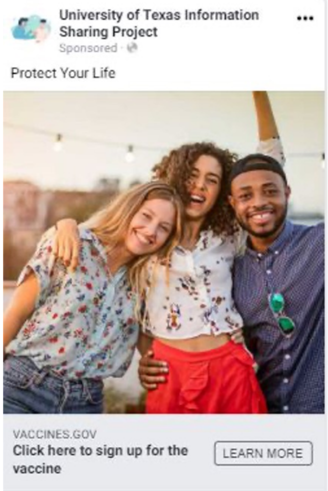
</td></tr>
<tr><td>US</td><td>B</td><td>Community</td><td>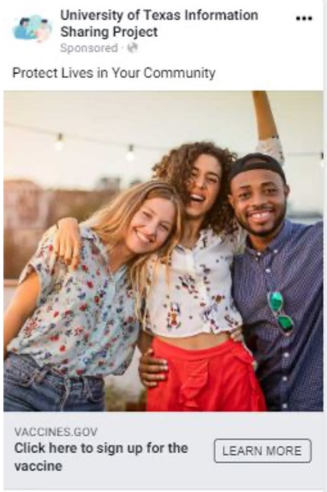
</td><td>B</td><td>Scientist</td><td>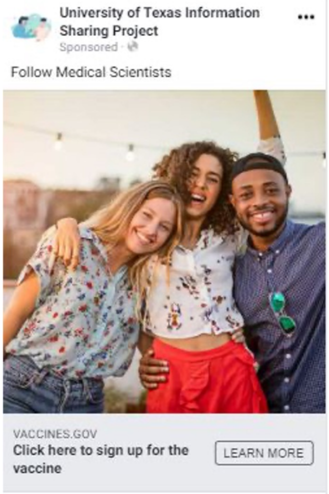
</td></tr>
<tr><td>US</td><td>B</td><td>Placebo</td><td>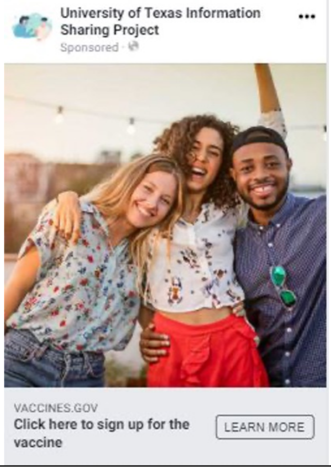
</td><td></td><td></td><td></td></tr>
</table>

Table S27. US: Group B Ads

6. Ad Placements

Table S28. Ad Placements by Country and Platform

	Facebook Reach	Instagram Reach	Facebook %	Instagram %
Brazil	380893	4363624	8.03	91.97
Russia	142303	1844146	7.16	92.84
SA	1528052	207254	88.06	11.94
Taiwan	1240019	526261	70.21	29.79
Turkey	2066468	717930	74.22	25.78
US	1642669	354521	82.25	17.75

7. Balance Test

Table S29. Balance Test

Country	Independence Test
Brazil	1
Russia	1
South Africa	0.7551
Taiwan	0.4546
Turkey	1
United States	0.9985

Draft

8. Language Translation of Treatment Message

8.1. Portuguese

Country Treatment Ad Content:

- Germany: Uma vacina amplamente usada foi desenvolvida na Alemanha
- US: Uma vacina amplamente utilizada foi desenvolvida nos Estados Unidos

Motivation Treatment Ad Content:

- Norms: 87% das pessoas foram vacinadas ou planejam ser vacinadas (de acordo com pesquisas da Morning Consult)
- Risk: A vacinação é 96% eficaz contra a hospitalização (incluindo da variante delta, de acordo com um estudo da Public Health England)

Action Treatment Ad Content:

- Family: Proteja vidas em sua família
- Community: Proteja vidas em sua comunidadeec
- Scientist: Siga os cientistas médicos
- Self: Proteja sua vida

Ad Elements:

- Headline: Clique aqui para se inscrever para a vacina
- Facebook Page Name: Projeto de compartilhamento de informações
- Vaccine signup link:
  https://vacinaja.sp.gov.br/?utm_source=portalutm_medium=banner-topoutm_campaign=Cadastro-Vacinaja

8.2. Russian

Country Treatment Ad Content:

- Germany: Одна из широко используемых вакцин была разработана в Германии.
- US: Одна из широко используемых вакцин была разработана в США.

Draft

Motivation Treatment Ad Content:

- Norms: 87% людей были вакцинированы или планируют вакцинацию (согласно опросам, проведенным Morning Consultom).
- Risk: Вакцинация эффективна на 96%, как профилактика против госпитализации (также включая дельта-вариант, что подтверждается в исследовании Public Health England)

Action Treatment Ad Content:

- Family: берегите свою семью
- Community: берегите жизни в своем общес
- Scientist: послушайте медицинских специалистов
- Self: берегите свою жизнь

Ad Elements:

- Headline: Нажмите здесь, чтобы зарегистрироваться на вакцинацию
- Facebook Page Name: проект обмена информацией цией
- Vaccine signup link:
  https://www.gosuslugi.ru/landing/vaccination

8.3. Traditional Chinese

Country Treatment Ad Content:

- Germany: 德國已研製出一種廣泛使用的疫苗
- US: 美國已研製出一種廣泛使用的疫苗

Motivation Treatment Ad Content:

- Norms: 87% 的民眾已接種或計劃接種疫苗（根據Morning Consult 的民意調查）
- Risk: COVID-19疫苗住院的保護力為96%（包括Delta變異株，根據英國公共衛生部的一項研究）

Action Treatment Ad Content:

- Family: 保護家人的生命

- Community: 保護社區中的生命
- Scientist: 信任醫學專家
- Self: 保護你的生命

Ad Elements:

- Headline: COVID-19疫苗接種院所
- Facebook Page Name: 信息共享平台
- Vaccine signup link: https://www.cdc.gov.tw/Category/List/hlrN4cZsF2Pe4C6DFhggqQ

8.4. Turkish

Country Treatment Ad Content:

- Germany: Oldukça yaygın kullanılan aşılardan biri Almanya'da üretiliyor.
- US: Oldukça yaygın kullanılan aşılardan biri Amerika Birleşik Devletleri'nde üretiliyor.

Motivation Treatment Ad Content:

- Norms: İnsanların 87%'si aşı oldu veya olmayı düşünüyor.
- Risk: Aşılar hastaneye yatma riskini 96% oranında azaltıyor (delta varyantı dahil).

Action Treatment Ad Content:

- Family: Aileni koru
- Community: Toplumu koru
- Scientist: Bilim insanlarına kulak ver
- Self: Virüsten korun

Ad Elements:

- Headline: Aşı olmak için şimdi kaydol
- Facebook Page Name: Doğru Bilgi Edinme Platformu
- Vaccine signup link: https://www.mhrs.gov.tr/

Draft
